# The Role of *Porphyromonas gingivalis* in Oral Carcinogenesis and Progression by Remodelling the Tumour Microenvironment: A Narrative Review

**DOI:** 10.3390/cancers17213478

**Published:** 2025-10-29

**Authors:** Katarzyna Starska-Kowarska

**Affiliations:** 1 Department of Physiology, Pathophysiology and Clinical Immunology, Department of Clinical Physiology, Medical University of Lodz, Żeligowskiego 7/9, 90-752 Lodz, Poland; katarzyna.starska@umed.lodz.pl; Tel.: +48-42-2725237; 2 Department of Otorhinolaryngology, EnelMed Center Expert, Drewnowska 58, 91-001 Lodz, Poland

**Keywords:** epithelial-to-mesenchymal transition (EMT), oral cancer, oral squamous cell carcinoma (OSCC), oral microbiome, periodontal pathogens, *Porphyromonas gingivalis*

## Abstract

**Simple Summary:**

*Porphyromonas gingivalis*, a Gram-negative anaerobic bacterium is the main inhabitant of the subgingival tissues in the oral cavity. Its presence has been associated with dysbiosis, periodontitis, a pro-inflammatory environment, and oral cancer due its secretion of virulence factors and ability to disrupt signalling pathways related to inter alia epithelial-to-mesenchymal transition (EMT) and cell-cycling. Numerous publications indicate that the periodontal disease caused by *P. gingivalis* and other oral microbiota is a significant risk factor for aerodigestive human cancers. This review provides a comprehensive overview of the role played by *P. gingivalis* in the initiation of carcinogenesis and the acquisition of an invasive phenotype of oral squamous cell carcinoma (OSCC); this has been attributed to its ability to initiate EMT, host immunosuppression, and inflammation-induced tissue damage, and to up-regulate cell proliferation and inhibit apoptosis by expressing or releasing virulence factors in the oral mucosa.

**Abstract:**

(1) Background: Oral squamous cell carcinoma (OSCC) is the most common type of head and neck malignancy worldwide. Despite the prevalence of modern diagnostic and prognostic techniques, late diagnosis and resistance to treatment still result in a low 5-year survival rate, high recurrence rate, and frequent malignant metastases. Increasing evidence indicates that bacteria of the oral microbiome, such as the Gram-negative anaerobic *Porphyromonas gingivalis*, may play a crucial role in the initiation and development of OSCC by inducing periodontitis. Indeed, epithelial-to-mesenchymal transition (EMT) and dysregulated immune response have been attributed to the activity of a dysbiotic microbiota. This comprehensive review examines the influence of *P. gingivalis* on oral carcinogenesis and progression, which has been associated with tumour microenvironment remodelling and the dysregulation of key signalling pathways related to epithelial-to-mesenchymal transition (EMT), cell-cycling, autophagy, and apoptosis. (2) Methods: The article reviews current literature on the possible role of *P. gingivalis* and induced dysbiosis, periodontitis and a pro-inflammatory environment as key mechanisms driving neoplastic epithelial changes and chemoresistance to anticancer agents in patients with OSCC; the research corpus was acquired from the Pub-Med/Medline/EMBASE/Cochrane Library databases. (3) Results: The identification of virulence factors and key mechanisms used by *P. gingivalis* to promote the development and progression of OSCC may support traditional diagnostic methods and factors related to treatment response and prevention of OSCC. (4) Conclusions: Emerging evidence suggests a possible association between periodontal bacteria and oral carcinogenesis. *P. gingivalis* may be an important potential target for future strategies aimed at treating oral cancer.

## 1. Introduction

### 1.1. The Characteristics of Oral Squamous Cell Carcinoma (OSCC)

Oral squamous cell carcinoma (OSCC), deriving from the oral mucosa, is the most common histological type of a heterogeneous group of malignant tumours known collectively as head and neck squamous cell carcinomas (HNSCC), themselves belonging to the head and neck cancers (HNC) [1,2]. The HNSCCs present a highly invasive clinical phenotype, which determines the clinical course of the disease, its progression and further development, mortality, treatment outcomes, and long-term prognosis, as well as local and nodal recurrence and patient survival. Studies indicate that HNSCC is two- to four-times more common in men than in women [3]; it is also the seventh most common cancer worldwide and accounts for nearly 4.5% of all human malignancies [GLOBOCAN 2020; gco.iarc.fr/today (accessed 31 August 2025)]. New cases of oral squamous cell carcinoma (OSCC) have been reported in over 377,713 patients, and they have resulted in 177,757 deaths [4,5]. Importantly, despite the development of modern therapeutic methods, the five-year/tumour-free survival of patients with OSCC has not changed over recent years.

Common risk factors for the development of HNSCCs, including OSCC, include smoking and alcohol consumption, as well as exposure to environmental pollutants and carcinogens. These factors are known to account for nearly 90% of cases of this type of cancer, as indicated by National Comprehensive Cancer Network (NCCN) data [6,7]. In addition, OSCC has been associated with the use of areca nut products, including betel quid and betel leaves, as well as slaked lime, poor oral hygiene and a diet low in vegetables [7]. Tobacco smoke is a major factor associated with the development of HNSCC, including OSCC, as it contains nearly 7000 toxic compounds with proven carcinogenic effects. The carcinogenic properties of its oncogenic components, such as benzo[a]pyrene, a key polycyclic aromatic hydrocarbon (PAH), nitrosamines (TNA) and N′-nitrosonornicotine (NNN), increase the risk of tumour initiation and progression and the formation of lymph node metastases and distant metastases. Alcohol acts as a co-carcinogen by causing epithelial atrophy, which can break down cell membrane lipids and thus facilitate the penetration of tobacco-derived carcinogens. Furthermore, the main metabolite of ethanol, acetaldehyde, is itself also a significant mutagen. The interaction of smoking and alcohol consumption increases the risk of HNSCC by up to 35-fold by inducing an epithelial-to-mesenchymal transition (EMT)-like phenotype [8,9,10].

Unfortunately, as many patients with HNSCC are diagnosed in a highly advanced stage, i.e., in WHO stages III and IV, modern diagnostic and therapeutic approaches based on surgical therapy and radiochemotherapy remain unsatisfactory. Indeed, the global overall survival or 5-year cancer-free survival parameters in the HNSCC population do not exceed 40 to 60% [11,12]. Data obtained from the Cancer Genome Atlas Network (TCGA) show that tobacco-related tumours are characterized by numerous mutations in four groups of genes: those regulating the cell cycle (*CDKN2A* and *CCND1*), those determining cell proliferation and survival (*TP53*, *HRAS*, *PIK3CA*, and *EGFR*), those controlling cell differentiation (*NOTCH1*), and a gene regulating the Wnt signalling pathway [3,13,14]. Mutations of proto-oncogenes, i.e., *c-myc*, *RAS* family genes (*KRAS*, *HRAS*, *NRAS*, *ERB-B*, *BRAF*, *HER-2*, *c-KIT*, *BCL-2*, *STAT3*), tumour suppressor genes called anti-oncogenes (*RB1*, *P53*, *PTEN*, *CDKN2A*, *INK4*), and genes controlling the pro-inflammatory tumour microenvironment, are also commonly observed in the course of cancers in this region [3,15].

Importantly, SCC of the oral cavity and the oropharynx can also be caused by infection with oncogenic strains of human papillomavirus (HPV), primarily HPV-16 and, to a lesser extent, HPV-18. The prevalence of these tumours has increased globally and now accounts for as many as 38–80% of new diagnoses of oropharynx squamous cell carcinomas (OPSCC). The carcinogenic factors in this type of HNSCC are the viral oncoproteins HPV16/18 E5, E6 and E7 [16,17,18,19]. The incorporation of HPV genomic DNA into the genetic material of the host epithelial cells results in molecular changes, leading to the initiation and development of cancer. Oncoproteins E6 and E7 degrade cell cycle regulatory proteins such as pRB and the tumour suppressor protein p53, thus disrupting intracellular signalling pathways regulating the cell cycle [9,20,21,22]. Importantly, the HPV16/18 E6 protein also deregulates the *c-myc* oncogene and activates transcription of the human telomerase catalytic subunit (hTERT). *c-myc* activity inhibits tumour cell immortalization and disrupts the function of CDKs, cyclins, and E2F transcription factors, and reverses the inhibitory effects of CDK p27^KIP1^ and p21^CIP1/WAF1^ [15,21].

According to TCGA, HPV-related cancers are characterized by alterations in the *PIK3CA*, *FGFR*, *DDX3X*, and *CYLD* genes [23]. HPV-related tumours may also experience amplification on chromosome 3q and loss of chromosomes 11q, 13q, 14q, 16p, and 16q [13,14]. Interestingly, HPV^+ve^ tumours most often affect young patients with higher social status, who, after diagnosis, have more satisfactory survival rates than HPV-negative patients, regardless of treatment modality [16]. Importantly, HPV-positive tumours are more frequently diagnosed in the early stages of the disease and are more responsive to chemotherapy and immune checkpoint inhibitors (ICIs) than non-HPV tumours. This justifies the use of the current de-escalation and de-intensified cure protocols used in clinical trials and highlights the need to improve the stratification strategy for patients with HPV^+ve^ HNSCC, particularly those with OSCC [24,25]. Due to the significantly molecular, morphological, and clinical differences between OSCC and tobacco-related HNSCC tumours, it has been proposed to change the traditional pTNM classification system to the new 8th edition AJCC/UICC system by the 2017 of the American Joint Committee on Cancer (AJCC). The treatment protocol for HPV-related tumours was associated with the proposal of de-intensification to achieve long-term improvement in treatment outcomes and ultimate cure [26]. Unfortunately, recent clinical trials (RTOG 1016 and De-ESCALaTE) have highlighted a more complex therapeutic challenge in patients with HPV-related tumours, as a large cohort of patients who received de-intensification treatment had significantly worse outcomes compared to those who received standard care [27,28]. Moreover, patients with HPV-positive multiregional primary OSCC and a second primary cancer in any head and neck location were found to have, among other things, a lower pT/pN grade compared to patients with a single primary tumour [29].

### 1.2. Porphyromonas gingivalis—A Keystone Pathogen in the Human Oral Microbiota

It is worth noting that approximately 15–20% of patients with oral cancer are neither heavy smokers nor alcohol drinkers and are not infected with carcinogenic types of HPV. In such cases, researchers consider inappropriate dietary habits and poor oral hygiene to play an important role, as well as periodontal diseases induced by infections with periodontal pathogens that promote oral dysbiosis; among these, the most abundant is *Porphyromonas gingivalis* [30,31]. Indeed, epidemiological studies and meta-analyses indicate that periodontal bacteria may play a role in the pathogenic mechanisms of oral cancer, especially OSCC [32,33,34].

Periodontal pathogens have been indicated as etiological factors for OSCC. They are believed to exert a multifaceted impact on oral carcinogenesis, cancer development, and metastasis by regulating specific molecular and cellular pathways leading to epithelial-to-mesenchymal transition (EMT), oral microbiome dysbiosis, and acquired immune evasion, leading to resistance to chemotherapeutic agents [35,36]. Unfortunately, despite widespread interest in the growth and expansion of oral microbiome-associated primary OSCC, little is understood of its biological mechanisms and the EMT induced by periodontal pathogens, as well as the molecular basis of the occurrence and development of oral cancer driven by periodontal pathogens.

Interestingly, research indicates that *P. gingivalis* may contribute to the increased risk of oral cancer in heavy alcohol drinkers by converting ethanol to acetaldehyde (ACH). ACH is the primary metabolite of alcoholic beverages and a well-known carcinogen. Acetaldehyde exposure can lead to DNA molecule damage, hyperproliferation, and enhanced cell cycling, as well as the activation of genomic changes, such as tumour suppressor mutations and increased proto-oncogene function [37,38]. The action of ACH may also be influenced by oral hygiene, a key factor known to protect periodontal tissues and the oral cavity from chronic inflammation and ethanol-associated tumourigenesis. Studies have shown that heavy drinkers and smokers with poor oral hygiene demonstrate an almost two-fold increase in local microbial salivary acetaldehyde production compared to healthy, non-drinking subjects [39,40]. Hence, in the case of poor oral hygiene, *P. gingivalis* may significantly increase the risk of oral cancer associated with ethanol consumption.

According to data from the enlarged Human Oral Microbiome Database, the oral mucosa harbours approximately 700 to 1000 different bacterial species [41]. The human body is also inhabited by over 100 trillion microbial cells living in symbiosis with their host [42]. These bacteria, by interacting with each other and other organisms, form a biofilm matrix that protects its creators from harmful environmental factors, such as antibiotics, as well as mechanical and chemical damage [43]. Thus, periodontal pathogens are able to evade immune-mediated defence mechanisms, allowing them to tolerate inflammation. Most members of the oral cavity microbiota are commensal bacteria living within the host tissues, but some species are odontogenic pathogens which may hasten the development of chronic oral diseases such as periodontitis/tooth loss and orodigestive cancers.

*P. gingivalis* is an anaerobic coccobacillus, a non-motile, and non-spore-forming Gram-negative bacterium; it occurs as various strains with different degrees of virulence [44]. *P. gingivalis* is one of the most-studied oral microorganisms that inhabits the subgingival sulcus of the human oral cavity and is considered a “keystone pathogen” in chronic periodontitis, leading to microbial dysbiosis and impaired host immune response. Recent clinical studies, as well as in vitro and animal models, clearly indicate that chronic inflammation caused by *P. gingivalis* infection is etiologically linked not only to a number of extraoral infection-related diseases such as diabetes, rheumatoid arthritis, or cardiovascular diseases, but also to orodigestive and pancreatic cancers [45,46,47]. Systematic reviews and meta-analyses of the prevalence of *P. gingivalis* and its association with oral cancer suggest that its presence may increase the risk of oral cancer development and periodontal disease by as much as 1.36 to 2.66 times [48].

Although *P. gingivalis* is most commonly associated with chronic inflammatory states, it can also be found in healthy individuals. Indeed, studies indicate that it may play a key role in oral carcinogenesis, regardless of periodontitis, even when it is present in low numbers [49,50]. Data show that *P. gingivalis* can inhabit oral niches in healthy individuals with a prevalence of approximately 25%, rising to 79% in individuals with periodontitis [51]. In oral cancer tissue samples, it was found to be significantly more prevalent in OSCC cases (>33%) than in normal gingival tissues [52].

The *P. gingivalis* bacterium possesses a plethora of virulence factors including lipopolysaccharide (LPS) [53], fimbriae (FimA/Mfa1) [54,55], RgpA/RgPb gingipains (cysteine proteinases) [56,57], outer membrane versicles (OMVs) [58], hemagglutinins (HA) [55,59,60], capsules [61,62], the citrullinating enzyme peptidylarginine deiminase (PPAD) [63,64], penta-acylated lipid (A-LPS), and a nucleoside-diphosphate-kinase (NDK) [65]. These virulence factors allow this opportunistic bacterium to evade recognition and destruction by the immune system, while also allowing it to tolerate, and even perpetuate, the inflammatory environment, thus increasing its chances of survival [66,67,68,69,70].

Moreover, *P. gingivalis* has been found to produce a wide repertoire of virulence factors which enable penetration of the gingivae, directly or indirectly resulting in rapid and significant periodontal tissue destruction, bone resorption, deep tissue invasion, and induction of host responses through cytokine production. Importantly, the level of aggression of biofilm components and the immune system complex are governed by interactions between pathogenic bacterial flora and host immune defence mechanisms; these also influence the function of protective host phenomena such as immunocompetent cells and determine the likelihood of periodontal tissue destruction or neoplastic metaplasia in the oral cavity [71,72].

Virulence factors are often expressed by the pathogen in response to changes in the external environment. However, their expression and disease mechanisms in various stages of periodontal disease and oral cancer remain unclear. To fully understand the specific function of each virulence factor and its mechanism in the pathogenesis of oral disease, they must first be inactivated and subjected to biochemical assessment and in vivo virulence research.

**Figure 1:**
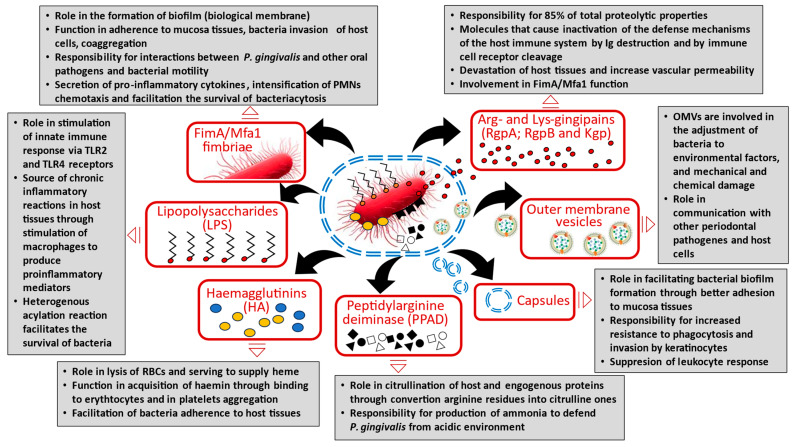
Virulence factors of *Porphyromonas gingivalis* and their function in human oral microbiota.

The virulence factors and host effectors produced by *P. gingivalis* are illustrated in Figure 1.

This review provides a comprehensive overview of the current understanding of the prevalence of the keystone oral pathogen *Porphyromonas gingivalis*, and the key specific molecular/cellular pathogenic mechanisms it uses in the initiation of carcinogenesis. It also examines the acquisition of an invasive phenotype by oral squamous cell carcinoma (OSCC) in response to various processes: EMT-induced by periodontal pathogens, host immunosuppression, inflammation-induced tissue damage, up-regulation of cell proliferation, and inhibition of apoptosis through bacterial virulence factors expressed or released in oral mucosa. It also discusses the role played by the periodontal pathogen community in regulating the initiation and development of OSCC and the introduction of novel molecular/cellular targets for oral cancer treatment. It also outlines the significant limitations of existing research available on this topic.

## 2. Materials and Methods

The corpus of research on the potential role of *Porphyromonas gingivalis* in oral squamous cell carcinoma (OSCC) is a broad one, encompassing a wide range of recent molecular and clinical studies. The works concern inter alia its role in the initiation of oral carcinogenesis and neoplastic development and the prognosis of patients with OSCC; the studies also touch on host microbiome mechanisms related to epithelial-to-mesenchymal transition (EMT), cell-cycle progression, apoptosis and autophagy, and dysregulated immune response. The present work is intended as a compendium of existing knowledge derived through in vitro research, animal models, and clinical studies, and is based on the latest publications and available data on this topic.

A comprehensive literature search in this narrative review was performed using the following inclusion criteria: (a) publications written in English describing the role of *P. gingivalis* in the process of carcinogenesis, invasiveness, treatment, and prognosis of OSCC; (b) in vitro studies; (c) studies based on animal tissues; (d) studies based on human tissues; (e) patients with confirmed diagnosis of squamous cell oral carcinoma; (f) clinical studies. All possible articles, whether original studies or reviews, were considered for this review, viz., cohort studies, retrospective studies and prospective studies. This review also provides an overview of the current understanding of the use of antimicrobial compounds in combating resistance to chemoradiotherapy. The final search (conducted 30 August 2025) included the most valuable and highest-rated peer-reviewed articles published in the past two decades (January 2000 to August 2025), all of which are available through the Pub-Med/Medline/EMBASE/Cochrane Library database. The databases were searched using the following keywords: “oral cancer”, “oral squamous cell carcinoma”, “periodontitis”, “periodontal disease”, “periodontal pathogens”, “epithelial-to-mesenchymal transition”, “epithelial-mesenchymal transition”, “EMT”, “carcinogenesis”, “diagnosis”, “prognosis”, “therapeutic or drug resistance”. Additional records were identified using cross-references. A manual search was also conducted combining “AND” or “OR” operators. Any obtained issues are discussed in order of increasing clinical plausibility.

The following exclusion criteria were applied: (a) unpublished articles or conference proceedings; (b) editorials, opinions, case reports, and letters to the editor; (c) studies in which the patient’s diagnosis is uncertain (e.g., lack of histopathological confirmation); (e) abstracts.

During data extraction, relevant information regarding the objectives, methods, results, and conclusions was collected from the extracted articles.

## 3. Results

### 3.1. In Vitro Models of Oral Cancer

#### 3.1.1. Activity of Epithelial-to-Mesenchymal Transition and Tumour Spread

Many recent in vitro studies have indicated potential links between infection by periodontal pathogens, such as *P. gingivalis*, and oral cancer [35,73,74,75,76,77,78,79,80,81]. Emerging evidence clearly suggests that the microbe is able to induce epithelial-to-mesenchymal transition (EMT), which can promote the initiation and/or development of OSCC. For example, an in vitro experiment by Ha et al. [73], in which OSCC cells were infected with *P. gingivalis* twice weekly for a total of five weeks, indicated a decrease in epithelial cell markers and a significant concomitant increase in mesenchymal markers, suggesting acquisition of EMT phenotype during oral carcinogenesis. Long-term *P. gingivalis* infection increased the expression of CD44 and CD133 antigens, well-known markers of stemness, which promoted the invasive features of infected cancer cells compared with uninfected controls. Furthermore, the *P. gingivalis*-infected OSCC cells demonstrated increased invasiveness, which significantly correlated with higher production of matrix metalloproteinases MMP-1 and MMP-10, driven by the release of IL-6 and IL-8, the key pro-inflammatory and pro-angiogenic cytokines.

Importantly, other studies have confirmed that infection with *P. gingivalis* increased the production and activity of MMP-1, -2, -7, -9, and -10 in cancer cells by modulating the IL-8/MMPs axis [35,74,75]. The mechanism of *P. gingivalis*-induced EMT was analyzed in heat-killed periodontal *P. gingivalis* separated from OSCC cell line cultures (H400) [35]. The bacteria were treated separately for eight days, and the expression of EMT-associated mesenchymal markers was assessed, together with the integrity of the cultured epithelial layer, the levels of cytokines TGF-β1, TNF-α, and EGF, and the ability of oral cells to migrate compared with unstimulated controls. Bacterial stimulation resulted in ∼6-fold upregulation of transcript levels of vimentin, Snail, and Twist and downregulation of E-cadherin relative to controls. Significant ∼2-fold upregulation of TNF-α, TGF-β1 and EGF was also detected.

Interesting conclusions regarding the carcinogenic mechanisms activated by *P. gingivalis* infection were presented by Gallimidi et al. [74], following co-incubation with tongue epithelium-derived oral cavity SCC cell lines SCC-25 and CAL 27. The findings indicate increased expression and functionality for the TLR2 receptors of OSCC cells, but not TLR4, leading to enhanced IL-6 production following treatment with ligands specific for these membrane molecules. These in vitro findings indicate that exposure of oral cancer cells to *P. gingivalis* resulted in the induction of additional cytokines, enzymes, and bioactive molecules involved in the proliferation, survival, and aggressiveness of oral squamous cell carcinoma, i.e., cyclin D1, TNF-α, MMP-9, and heparanase. Interestingly, Cho et al. [75] analyzed the anti-invasive effect of acetylshikonin, a flavonoid with anti-inflammatory activity, on *P. gingivalis*-infected YD10B oral cancer cells. It was found that *P. gingivalis* infection increased the invasiveness of YD10B OSCC cells, and that acetylshikonin significantly inhibited this invasiveness by inhibiting IL-8 production and IL-8-dependent MMP release.

Many studies of the role of *P. gingivalis* infection in EMT induction have examined changes in the expression of key carcinogenic regulators and crucial pathways at the molecular level in infected oral epithelial cells. For instance, an analysis of the expression and activation of main EMT mediators during long-term *P. gingivalis* infection in vitro by Lee et al. [76] noted that human oral primary epithelial cells (OECs) enhance tumourigenic properties and acquire an EMT phenotype when *P. gingivalis* was present in oral mucosa. Cells infected for 120 h with *P. gingivalis* were found to be predisposed to neoplastic transformation by EMT; this greater predisposition was associated with a higher phosphorylation status of glycogen synthase kinase 3 beta (p-GSK3β), an important regulator of EMT, and increased expression of EMT-related transcription factors, i.e., Slug, Snail, ZEB1, Twist, and vimentin, at both the protein and mRNA levels. Furthermore, the OECs demonstrated a significant decrease in the expression of the adhesion molecule E-cadherin and the loss of its membrane localization, together with β-catenin. These phenomena were accompanied by a marked increase in the levels of matrix metalloproteinases MMP- 2, -7 and -9. Interestingly, a study by Sztukowska et al. [77] showed that infection of gingival epithelial cells by *P. gingivalis* expressing FimA fimbriae led to higher activity and nuclear localization of the transcription factor ZEB1, which controls epithelial–mesenchymal transition. *P. gingivalis* strains lacking FimA were unable to induce ZEB1 expression. Furthermore, epithelial cells with higher levels of ZEB1 demonstrated increased expression of mesenchymal markers, including vimentin and MMP-9, and intensified motility and migration into matrigel.

In addition, primary epithelial cells infected with *P. gingivalis* can also acquire a mesenchymal phenotype through the activity of GSK3β; this factor increases PI3K/Akt pathway activation, resulting in the subsequent upregulation of associated transcription factors [78]. This in turn increases the expression of mesenchymal markers via the loss of E-cadherin expression, accompanied by the non-canonical activation and subcellular localization of β-catenin. In another interesting study, Hoppe et al. [79] describe the molecular effect of PI3K/Akt axis activation on EMT induction. The authors observed that *P. gingivalis* induced the production and secretion of TGF-β1 into the pericellular matrix, thus leading to an Akt-dependent mesenchymal transformation of immortalized OKF6 oral keratinocytes. Another possible mechanism of pathogen-induced EMT in oral epithelial cells was outlined by Liu et al. [80]; the authors found *P. gingivalis* to deliver low-molecular-weight tyrosine phosphatase (Ltp1) to gingival epithelial cells, which then takes part in the dephosphorylation of PTEN, a negative regulator of PI3K/Akt signalling, leading to loss of PTEN stability. A decrease in the activity of dephosphorylated PTEN was associated with increased cell migration and partial induction of EMT through upregulation of the transcription factor ZEB2.

Interesting findings were also acquired by Utispan et al. [81], who analyzed the effect of *P. gingivalis* infection of HNSCC cells on tumour proliferation and invasiveness. The researchers examined human primary (HN18, HN30, and HN4) and metastatic (HN17, HN31, and HN12) HNSCC cell lines treated with the monocyte THP-1 and LPS-induced macrophage-conditioned medium (CM). Macrophages in the proposed study system were characterized by increased expression of IL-6 and CD14. Furthermore, *P. gingivalis* LPS stimulated the production of NO by macrophages, and increased its secretion, although TNF-α production was reduced. LPS-induced macrophage CM inhibited the proliferation of HN4 cells. Interestingly, LPS-induced CM macrophage promoted invasion by all HNSCC cell lines.

**Figure 2:**
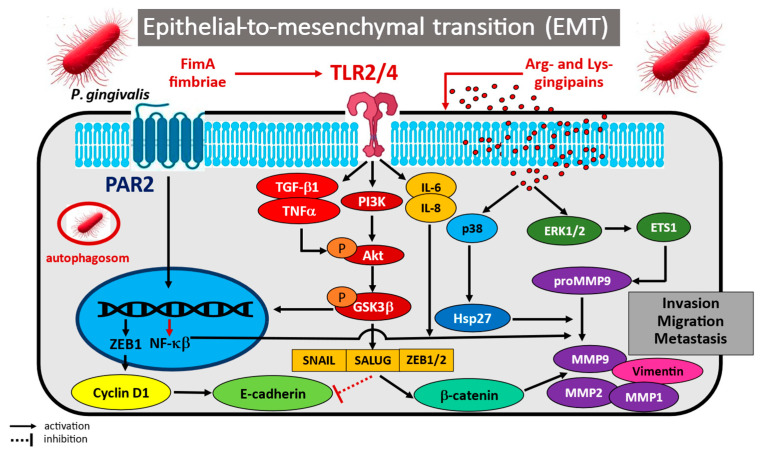
The role of *Porphyromonas gingivalis* in the initiation and progression of oral cancer, including pathogen-induced intracellular signalling pathways. The colonization by *P. gingivalis* induces epithelial-to-mesenchymal transition (EMT) with its virulence factors, i.e., fimbiare (FimA) and Arg- and Lys-gingipains (HRgpA and RgpB). The invasiveness of *P. gingivalis*-infected OSCC cells was characterized by the higher production of matrix metalloproteinases MMP-1, -2, -7,-9, and -10, which was stimulated by the release of IL-6 and IL-8, ERK1/2, p38, and Hsp27. The oral cells exhibited higher levels of cytokines TGF-β1, TNF-α, and EGF, as well as reduced integrity of the cultured epithelial layer. In addition, the TLR2 and TLR4 receptors in OSCC also demonstrated altered expression and functionality. The cells infected with *P. gingivalis* underwent neoplastic transformation following an increase in the expression of p-GSK3β and the EMT-related transcription factors Slug, Snail, ZEB1, Twist, and vimentin. The activity of GSK3β led to increased PI3K/Akt pathway activation, and subsequent upregulation of transcription factors. These intracellular changes facilitate the loss of E-cadherin expression and nucleocytoplasmic accumulation of β-catenin. *P. gingivalis* infection affected the activity of signalling cascade ERK1/2-ETS1, p38/HSP27, and protease activated receptor PAR2 and transcription nuclear factor NF-κB.

The potential mechanisms of *Porphyromonas gingivalis* in pathogen-induced epithelial-to-mesenchymal transition (EMT) are illustrated in Figure 2.

#### 3.1.2. Pro-Inflammatory Effects and Regulation of the Immune Response

Infection of the oral mucosa by *P. gingivalis* can also support neoplastic development in the oral cavity by influencing inflammation-related pathways, leading to escape from the immune system response [82,83]. For example, *P. gingivalis* has been implicated in the activation of key intracellular pathways related to transcription factor NF-κB and p38MAPK activation [82,83]. Groeger et al. [82] showed that the bacterial biofilm at the gingival margin can induce a host immune reaction. An analysis of signalling cascade activation in primary epithelial cells and oral cancer cell lines identified numerous genes that may be responsible for the observed immune imbalance. The authors demonstrated that *P. gingivalis* infection leads to increased RNA expression of genes involved in the antibacterial immune response, including the NF-κB signalling pathway, IKBKB, TLR signalling and IRF5, and MAPK pathway family kinases (*JUN*, *MAP2K4*, *MAPK14*, and *MAPK8*), thereby enhancing the pro-inflammatory immune response in primary and malignant oral epithelial cells SCC-25. Similar observations were noted by Milward et al. [83], who investigated the effect of *P. gingivalis* infection on activation of Toll-like receptors 2, 4, and 9 and NF-κB signalling pathway components, as well as their related genes, in oral epithelial cells and H400 oral epithelial cell line. Researchers confirmed that infection with pathogenic bacteria enhances NF-κB nuclear translocation and increases the expression of its dependent genes, including the following cytokines/chemokines: *TNF-α*, *IL-1β*, *IL-8*, monocyte chemoattractant protein-1 (*MCP-1/CCL2*), and *GM-CSF*, as well as heme oxygenase-1 (*HM0 × 1*), lysyl oxidase (*LOX*), superoxide dismutase 2 (*SOD2*), *CCL20*, calprotectin, and S100 calcium binding protein A8/A9 (*S100A8/A9*) components. Importantly, in the oral epithelium, the pro-inflammatory effects of TLRs/NF-κB/p38MAPK signalling pathway activation may play an important role in the induction of oxidative stress and cellular DNA damage, key phenomena in the process of carcinogenesis [84].

In addition, the activation of immunocompetent cells via specific receptors, such as squamous carcinoma/transformed cells and gingival keratinocytes, results in chronic inflammation that frequently precedes the development of human cancers. Studies clearly indicate that upregulated expression of immune regulatory B7 homologue 1 (B7-H1) receptor and a new dendritic cell B7 family member (B7-DC) receptor present on cancer and oral epithelial cells may lead to anergy and apoptosis of activated T cells. This altered immune cell activation has been associated with the interference of B7-H1/B7-DC receptors on neoplastic cells with PD1 receptor present on tumour-infiltrating lymphocytes (TILs); this is believed to block the PD1-induced cytotoxic response against cancerous epithelial cells. It is also one of the mechanisms by which cancer cells escape host immune surveillance. For example, Groeger et al. [85] assessed the in vitro expression of B7-H1 and B7-DC receptors on squamous cell carcinoma cell lines SCC-25 and BHY and primary human gingival keratinocytes (PHGK) following infection with two virulent strains of *P. gingivalis* (W83 and ATCC 33277^T^). It was found that infection of the epithelium caused a significant increase in the expression of B7-H1 and B7-DC receptor mRNA, and that the mean intensity of fluorescence for these receptors was more than six-fold (B7-H1) and eight-fold (B7-DC) higher in SCC-25 cancer cells, and more than eight-fold (B7-H1) and five-fold (B7-DC) higher in PHGK cells. These observations were also confirmed in a later study by the same group who indicated that the B7-H1 receptor, also known as programmed cell death ligand (PD-L1), plays an important role in the mechanism leading to immune evasion [86]. The activity of the B7-H1 receptor, an immune-signalling molecule that mediates the regulation of T cell activation and tolerance, negatively impacts T cell function and survival. Importantly, after stimulation with membrane fractions of *P. gingivalis*, human squamous cell carcinoma cells and primary gingival keratinocytes were found to significantly increase PD-L1 protein expression. Interestingly, stimulation of tumour/epithelial cells with cytosolic proteins and LPS did not induce such changes. The anergy and tolerance of the induced immunocompetent cells led to evasion of the immune response in the studied systems. Wang et al. propose an alternative mechanism that can lead to increased pro-inflammatory IL-6 production and signal transducer and activator of transcription 3 (STAT3) activation in oral epithelial cells infected with *P. gingivalis* [87,88]. They note that IL-6 production resulted from the activation of the Janus kinase 2 (JAK2) and glycogen synthase kinase 3 beta (GSK3β) pathways, key mediators of the suppression of innate immune system response to bacterial stimuli; these pathways are also responsible for cell cycle activation, enhanced cell differentiation and cell mobility, inhibition of apoptosis, and greater cancerogenic epithelial cell migration. The researchers also indicate that phosphor-inactivation of the GSK3β molecule also led to increased expression of pro-tumourigenic IL-10, which inhibits the immune response and facilitates escape from immune surveillance in epithelial cells exposed to bacterial stimuli [87].

Furthermore, regulation of the host’s innate immune response was found to be mediated by the induction of reactive oxygen species (ROS) activated by oral pathogens, with the mechanisms related to JAK2 phosphorylation and increased secretion of pro-inflammatory IL-6 and IL-1β. In the studied systems, ROS-mediated JAK2 phosphorylation led to the phosphor-activation of c-Jun protein kinase (JNK) and the transcriptional regulator c-Jun. Interestingly, pharmacological inhibition or siRNA-mediated gene silencing of JNK or c-Jun were found to reduce the cytokine levels elevated by *P. gingivalis*. Hence, it appears that ROS-mediated JAK2 activation is necessary for *P. gingivalis*-induced inflammatory cytokine production, and the JNK/c-Jun signalling axis leads to ROS-dependent regulation of IL-1β and IL-6 production [88].

Key indicators of the role of *P. gingivalis*-induced chronic inflammation in the course of chronic periodontitis are epigenetic changes, such as chemical changes in DNA and regulatory proteins, leading to chromatin remodelling, as well as alterations in gene transcription and cytokine profile. Indeed, several publications have demonstrated an association between the presence of pathogens such as *P. gingivalis* in gingival tissue and the maintenance of inflammatory processes [89,90,91,92]. For example, Diomede et al. [89] examined the association between epigenetic modulations and periodontitis using human periodontal ligament stem cells (hPDLSCs) in vitro. After stimulation with *P. gingivalis* LPS, the hPDLSC cells exhibited altered the expression of proteins associated with DNA methylation and histone acetylation, such as DNMT1 and p300, respectively, and the inflammatory transcription factor NF-κB. Specifically, *P. gingivalis* LPS treatment led to the simultaneous inhibition of DNMT1 DNA methylase activity, and activation of p300 histone acetyltransferase and NF-kB function in hPDLSCs. Similar conclusions were presented by Martins et al. [90], who observed that bacteria are able to induce epigenetic modifications in oral epithelial cells mediated by histone modifications. The researchers found that the presence of dysbiosis in vivo triggers histone acetylation and decreased DNMT1 activity. Furthermore, the exposure of oral epithelial cells to LPS in vitro was also associated with histone modifications, activation of transcriptional coactivators such as p300/CBP, and accumulation of NF-κB. Also, the activation of Toll-like receptors 1, 2, and 4 and nucleotide-binding oligomerization domain protein 1 (NOD1) was found to induce histone acetylation and play a role in regulating the innate immune system. Interesting observations were also obtained from primary cultures of gingival fibroblasts and keratinocytes (HaCaT) infected with *P. gingivalis*, as reported by de Camargo Pereira et al. [91]. The cells were exposed to LPS for 24 h to assess the expression of epigenetic enzymes, viz. DNA methyltransferase 1 (DNMT1), DNA methyltransferase 3a (DNMT3a), Jumonji domain-containing histone demethylase 3 (JMJD3), and ubiquitously transcribed tetratricopeptide repeat, X chromosome (UTX). The aim was to compare the activity of genes regulating epigenetic changes between healthy individuals and those affected by periodontitis. Unlike previous studies, no significant differences in gene expression were noted between the two groups of gingival samples. However, LPS was found to decrease the expression of all studied genes in HaCaT cells, but no modulation was observed in gingival fibroblasts.

Other authors have also reported a variety of miRNA-mediated cellular responses to *P. gingivalis*. Olsen et al. [92] found some miRNA species (miRNA-128, miRNA-146, miRNA-203, and miRNA-584) to play a regulatory role in the innate immune system, suggesting that these molecular signatures also have therapeutic potential. The authors also indicate that periodontal bacteria LPS may influence the nature of host miRNAs and their mRNA targets. In summary, these publications support the hypothesis that *P. gingivalis* infection may induce pro-inflammatory effects and lead to evasion of the immune response and epigenetic changes. These findings may offer an insight into the mechanisms involved in the induction of chronic periodontitis and its subsequent carcinogenesis. The identified genetic changes in key pro-inflammatory intracellular pathways and/or epigenetic changes in DNA methylation and histone acetylation may constitute potential targets for the diagnosis and treatment of periodontal disease; however, further research on these phenomena is necessary.

**Figure 3:**
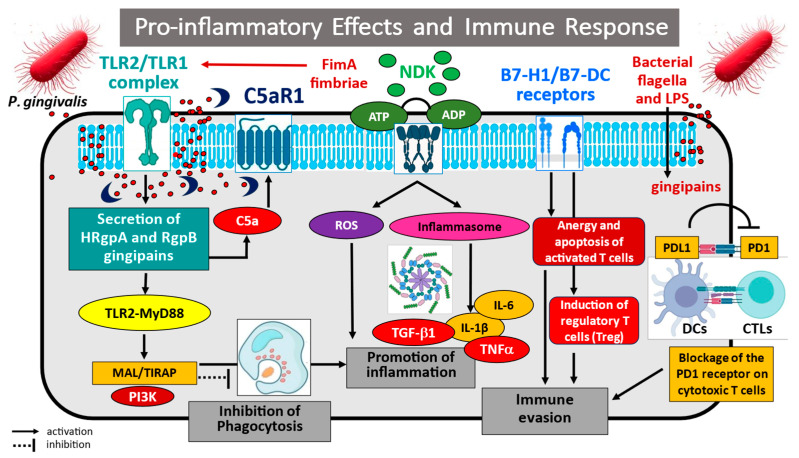
*P. gingivalis* infection triggers pro-inflammatory mechanisms and regulates the immune response to biofilm pathogens. *P. gingivalis* induces the overexpression of B7-H1 and B7-DC receptors in oral cells; these are responsible for chronic inflammation through the increased production of IL-1β, IL-6, IL-8, and TNF-α. Expression of B7-H1 receptor inhibits effector cells by inducing regulatory T cells (Treg), anergy, and apoptosis of activated immune cells. Bacterial flagella and LPS induce cancer-promoting inflammatory reactions. Secreted gingipains also contribute to the regulation of the relationship between cytotoxic T lymphocytes and antigen-presenting cells, such as dendritic cells. The B7-H1 receptor is upregulated on cancer cells and interferes with the PDL1 receptor on TILs, blocking the PD1-like cytotoxic T cells. The cell surface molecule TLR2-TLR1 complex induces gingipain secretion, which influences complement C5 to generate the C5a ligand for the C5aR1 receptor. The pathogen induces C5aR1-TLR2 signalling in neutrophils and macrophages, which separates the host protective TLR2-MyD88 pathway from the TLR2-MyD88-adaptor-like (MAL, also known as TIRAP)-PI3K pathway, which blocks phagocytosis and promotes inflammation. The activity of nucleoside-diphosphate-kinase (NDK), allowing ATP activation of P2X7 receptors, also contributes to the formation and release of reactive oxygen species (ROS) and activation of the inflammasome, and thus increased secretion of the pro-inflammatory cytokines IL-1β, TNF-α, TGF-β1, and IL-6.

The potential mechanisms of *Porphyromonas gingivalis* in pathogen-induced immune evasion are illustrated in Figure 3.

#### 3.1.3. Regulation of Cell Cycling and Proliferation

A considerable body of evidence indicates that periodontopathogens, especially *P. gingivalis*, may be responsible for uncontrolled cell division and proliferation in oral squamous cell carcinoma (OSCC) [93,94,95,96,97,98]. Chang et al. [93] examined the molecular mechanisms responsible for increased OSCC cell proliferation in an in vitro model of a multiplicity of *P. gingivalis* infection with the Tca8113 cell line. It was found that the infected neoplastic cells exhibited significantly faster division than the healthy control cells; the infected cells were also characterized by a greater percentage of cells in S phase and a lower percentage in G1 compared to controls. The researchers also identified certain molecular mechanisms that may play a role in the activation of cell division, with the most likely being increased activity of activator protein 1 (AP-1; c-Jun, and c-Fos), miR-21, and its target gene *cyclin D1*, as well as decreased expression of programmed cell death 4 (PDCD4). These studies conclude that *P. gingivalis* infection may promote the proliferation of oral cancer cells by activating the miR-21/PDCD4/AP-1 negative feedback signalling pathway.

Interesting observations regarding the infection of OSCC cancer cells with *P. gingivalis* were also made by Hoppe et al. [94], who analyzed the expression of oncogenic *α-defensin* genes, i.e., antimicrobial peptide components of the innate immune response, and the activity of the oncogenic EGFR pathway. It was found that incubation increased the expression of α-defensins and enhanced cell proliferation; this was caused by increased activity of the EGF/EGFR pathway, which was activated by defensins acting as EGFR ligands.

Geng et al. [95] analyzed the effect of chronic *P. gingivalis* infection on carcinogenesis in OSCC using a different in vitro model in which human immortalized oral epithelial cells (HIOEC) were exposed to *P. gingivalis* infection for 5 to 23 weeks. Cell proliferation and invasion were studied using microarrays and proteomic analysis. The findings indicate that long-term exposure to *P. gingivalis* infection led to a shift in cell phenotype toward mesenchymal characteristics, increased cell proliferation, induced the S phase, and promoted cell migration and invasiveness. Importantly, the authors also demonstrated that tumour-related genes, such as *NNMT*, *FLI1*, *GAS6*, lncRNA *CCAT1*, *PDCD1LG2*, and *CD274*, were key regulators of neoplastic transformation in response to a low multiplicity of oral pathogen infection. The results indicate that chronic *P. gingivalis* infection may be a significant potential risk factor for oral cancer, and the identified biomarkers may represent promising targets for prevention and treatment.

Zhou et al. [96] analyzed the potential signalling pathway involved in tumourigenesis in the oral cavity. The investigators indicated that *P. gingivalis* activation of β-catenin signalling, a key pathway controlling cell proliferation and tumour development, can lead to the induction of gingipain-dependent proliferation of gingival epithelial keratinocytes (TIGKs). Indeed, the findings indicate that the W83, ATCC 33277 *P. gingivalis* strains required gingipain proteolytic activity to induce nuclear translocation, upregulation of the β-catenin-dependent TCF/LEF promoter, and positive regulation of non-canonical β-catenin function. Furthermore, components of the β-catenin-destroying complex, viz. Axin1, adenomatous polyposis coli (APC) and GSK3β, were also subjected to proteolysis by the gingipain virulence factor. The results indicate that *P. gingivalis* infection of the oral epithelium induces non-canonical activation of β-catenin and dissociation of the β-catenin-destroying complex; these processes may support the development of a proliferative phenotype.

Several studies have discussed the role of *P. gingivalis* 33277 and YPF1 infection in accelerating cell cycling in gingival epithelial cell culture. A proteomic analysis of infected primary gingival epithelial cell (GEC) cultures by Kuboniwa et al. [97] found that infection stimulates changes in the level and phosphorylation status of proteins controlling the cell cycle. It was also observed that *P. gingivalis* regulated various intracellular pathways, including the action of cyclins and CDKs, p53 protein, and PI3K/Akt signalling molecules. Increased phosphorylation of CDK2 and accumulation of Cyclin A were noted. Additionally, both the level and the activity of p53 were decreased. The studied systems exhibited increased proliferation of gingival epithelial cells infected with *P. gingivalis*, resulting from accelerated cell progression into the S and G2 phases, although this was dependent on the presence of long fimbriae (FimA).

Similarly, Pan et al. [98] demonstrated that the presence of *P. gingivalis* in periodontal and gingival tissue contributes to the development and progression of periodontal disease. In the IHGE model of human gingival epithelial cell invasion, it was found that *P. gingivalis* infection can induce cell growth and division by accelerating the G1/S phase of the cell cycle, with cyclin D1 and cyclin E mRNA levels being significantly elevated within hours of invasion. Hence, it appears that the studied bacteria can manipulate the cell cycle of the host, allowing for bacterial survival and expression of virulence factors in the host.

However, this has been contradicted in some studies. An analysis of the proliferative activity, cell cycle, autophagic response, and reactive oxygen species (ROS) generation in oral cancer cells invaded by *P. gingivalis* FCD 381 strain by Cho et al. [99] indicated that infection inhibited oral cancer cell proliferation by inducing cell cycle arrest in the G1 phase. Furthermore, the presence of the pathogen inhibited the expression of cyclin D1 and CDK4 in cancer cells, and increased CDK inhibitor p21^CIP1/WAF1^ level compared to uninfected control cells. The observed inhibition of proliferation was attributed to increased macroautophagy in infected cells, induced by the formation of ROS. Indeed, several authors have proposed increased macrophage counts and inhibition of mitochondrial- and membrane (NADPH-oxidase)-derived ROS generation as key mechanisms facilitating the survival of pathogens in the oral epithelial environment [100,101].

Studies indicate that *P. gingivalis* infection may contribute to the inhibition of ROS production in human primary oral epithelial cells, thus enabling the growth and survival of periodontal pathogens. Choi [100] reports that primary gingival epithelial cells (GECs) produce cellular ROS upon stimulation by extracellular ATP (eATP) via P2X_7_ receptor signalling in conjunction with NADPH oxidase activation. Invasion of GECs by *P. gingivalis* activated antioxidant glutathione expression in response and modulated eATP-induced production of cytosolic and mitochondrial ROS generated by the P2X_7_/NADPH oxidase interactome. Furthermore, the researchers found the oxidative stress response in GECs to be blocked by the secretion of the *P. gingivalis* effector nucleoside diphosphate kinase (NDK), and an increase in the antioxidant mitochondrial uncoupling protein 2 (UCP2).

Similarly, Roberts et al. [101] report that *P. gingivalis* is able to survive the effects of extracellular eATP/P2X7 signalling in host cells and the production of ROS and NADPH oxidase (NOX) in primary GECs. As such, it appears that within 24 h of infection, *P. gingivalis* infection can support the inhibition of the NOX2 pathway by reorganizing the localization and activation of cytosolic p47phox, p67phox, and Rac1, and reducing myeloperoxidase (MPO) production. Furthermore, the presence of the bacteria in the oral epithelium was associated with increased expression of glutathione synthetase and glutathione reductase. This suggests that these mechanisms used by *P. gingivalis* allow periodontal pathogens to evade antimicrobial defences and successfully survive in human epithelial tissues.

Lee et al. [102] provide an interesting insight into the mechanisms enabling *P. gingivalis* to survive and replicate in its primary intracellular niche in the oral cavity. The researchers analyzed serial sections of infected GECs and visualized double-membraned, endoplasmic reticulum-rich autophagosome vacuoles, where *P. gingivalis* bacteria were present. Importantly, only cytosolic *P. gingivalis* were found to undergo lysosomal destruction, which was associated with the expression of the ubiquitin-binding adaptor proteins NDP52 and p62. The vacuolar fraction of the bacterium was protected from degradation in ER networks. The results suggest that *P. gingivalis* uses a novel mechanism of autophagy (pro-bacterial autophagy) in GECs to establish an effective replicative niche and survive in the oral mucosa.

The mechanisms described above, and the specific molecules through which *P. gingivalis* regulates the proliferation of oral epithelial and oral cancer cells, may represent promising targets against pathogen-induced tumourigenesis. Unfortunately, the body of evidence is ambiguous, and further in-depth analyses are required to identify new mechanisms by which *P. gingivalis* can accelerate cell proliferation. These ambiguities concern the status of *P. gingivalis*, the specificity of the tissues studied and methodologies, as well as the influence of inter alia the pathologic periodontal pathogen community and their relationships in the tumour epithelial microbiome.

**Figure 4:**
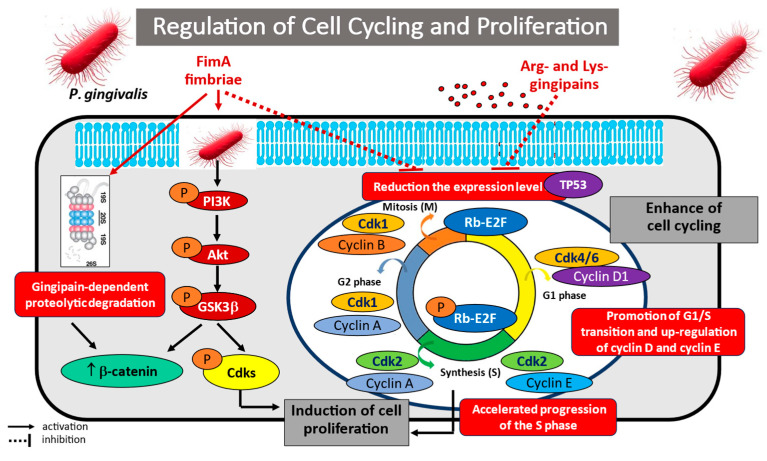
*P. gingivalis* infection regulates cell cycling and cell proliferation through activation and phosphorylation of cyclin-dependent kinases (CDKs) and reduces TP53 expression through the possession of fimbriae (FimA). This periodontal pathogen accelerates progression through the S phase of the cell cycle by preventing p53 tumour suppressor gene activity and PI3K activation. Lowering TP53 expression promotes G1/S transition and upregulation of cyclin D1 and Cyclin E. The proliferation rate is also enhanced by modifying the expression of the oncogenic α-defensin gene and EGFR-dependent signalling. Also, it is possible that *P. gingivalis* may influence carcinogenesis through its role in the activation of β-catenin proteolysis by the non-canonical gingipain-dependent pathway.

The potential mechanisms of *Porphyromonas gingivalis* in regulating cell cycling and proliferation are illustrated in Figure 4.

#### 3.1.4. Regulation of Apoptosis

Several recent publications have noted the involvement of *P. gingivalis* in the regulation of apoptosis in gingival epithelial cell and oral cancer cell cultures, indicating that it may also regulate periodontitis and carcinogenesis. Indeed, suppression of mitochondrial-dependent apoptosis has been found to be an important mechanism used by *P. gingivalis* to ensure its survival and persistence in the epithelial tissues of the oral cavity. Infection of the oral cell epithelium by *P. gingivalis* appears to promote the activation of key antiapoptotic pathways such as JAK1/STAT3, PI3K/Akt (protein kinase B), and Akt/FOXO1 signalling, as well as ATP-dependent apoptosis. Moreover, virulent factor LPS of *P. gingivalis* contains 2-keto-3-deoxyoctonate (KDO), which inhibits the intrinsic mitochondrial apoptosis of epithelial cells [103,104,105,106,107,108,109,110,111,112].

For example, Mao et al. [103] described the mechanisms by which *P. gingivalis* ATCC 33277 and A7A1-28, ATCC 49417, and W83 strains modulated intrinsic apoptotic pathways in oral epithelium. Infection inhibited chemically induced apoptosis in primary cultures of gingival epithelial cells (GECs) by blocking the activation of the effector caspase-3, regardless of the presence of fimbriae (FimA). The epithelial cells achieved an antiapoptotic phenotype by regulating mitochondrial apoptotic death pathways; this was associated with the promotion of JAK1, STAT3, and survivin phosphorylation and activation. Furthermore, JAK1 stimulation was accompanied by an increase in pPI3K/Akt signalling activity, which could be inhibited using siRNA molecules. Therefore, the induction of intracellular mechanisms promoting epithelial cell survival prevents programmed host cell death, thus facilitating the survival of *P. gingivalis* in gingival epithelial cells. Similar results were reported by Yilmaz et al. [104], who confirmed that bacteria are able to survive longer in infected primary gingival epithelial cells (GECs) by inhibiting the externalization of phosphatidylserine on the cell surface; this mechanism, requiring caspase activation, protects infected cells from apoptosis. The authors demonstrate that infection with *P. gingivalis* ATCC 33277 can block the depolarization of the mitochondrial transmembrane potential blockage of mitochondrial membrane permeability and release of cytochrome c, thus inhibiting DNA fragmentation and the apoptosis of infected GECs. Furthermore, during *P. gingivalis* infection, protein kinase B/Akt phosphorylation and regulatory activation of the PI3K/Akt signalling pathway may inhibit the inflammatory response while simultaneously promoting host cell survival.

However, not all studies confirm that *P. gingivalis* infection results in increased PI3K/Akt signalling pathway activity. Nakayama et al. [105] report that a living periodontal pathogen, via gingipain, can decrease Akt activity by reducing phosphorylation at both Thr-308 and Ser-473. Furthermore, Bad, PI3K, and glycogen synthase kinase 3α/β, the mammalian target of rapamycin, also underwent dephosphorylation, leading to their inactivation. The use of gingipain-specific inhibitors and a gingipain-deficient *P. gingivalis* mutant KDP136 revealed that the gingipains and their protease activities were essential for the inactivation of the PI3K regulatory subunit p85α and Akt. It is worth noting, however, that these effects of *P. gingivalis* infection were observed in wild-type bacteria, which induced low production of phosphatidylinositol 3,4,5-triphosphate by PI3K. This was due to the fact that PI3K failed to transmit homeostatic extracellular stimuli to intracellular signalling pathways, leading to the dysregulation of PI3K/Akt-dependent cellular functions and destruction of epithelial barriers.

*P. gingivalis* is also able to enhance the antiapoptotic activity of gingival epithelial cells (GECs) by regulating Bcl-2/Bad/Bax activity. This has been confirmed in various studies examining the activities of pro- and antiapoptotic molecules and signalling pathways known to regulate mitochondrial-dependent apoptotic process [78,106]. For example, Yao et al. [78] showed that GECs infected with *P. gingivalis* are protected against apoptosis by negative regulation of the proapoptotic Bcl-2 family members Bad and Bax, through phosphorylation, sequestration, and translocation into the cytosol; all of these phenomena are dependent on the activity of the pPI3K/Akt pathway. Caspase-9 activity was also blocked. These mitochondrial pathways represent an important mechanism for protecting host cells against induced cell death, and thus ensuring the survival of *P. gingivalis* in the gingival epithelium. Nakhjiri et al. [106] describe the effects of invasion by *P. gingivalis* in primary GEC culture. Interestingly, following transient induction of DNA fragmentation, prolonged incubation was found to inhibit apoptosis. The antiapoptotic effect was reflected by the stimulation of Bcl-2 at the transcriptional level and a reduction in Bax activity.

A very interesting finding is that *P. gingivalis* appears to be able to control apoptosis in infected tissue by activating microRNA, which influence the activity of pro- and antiapoptotic regulatory molecules. Moffatt et al. [107] analyzed the ability of *P. gingivalis* strain 33277 to regulate the expression of several key miRNAs, including miR-149, -203, -205, -181a, -1308,-107, -26a, -221, -200, and -1826 in GECs. It was found that microRNA-203 activation appears to be a key element in the anti-apoptosis cascade, with four-fold higher expression in infected cells compared to uninfected controls, as well as five-fold inhibition of suppressor of cytokine signalling 3 (SOCS3) and two-fold inhibition of SOCS6. Further analysis showed that the inhibition of signal transducer and activator of transcription 3 (STAT3), a downstream target of SOCS, inhibited apoptosis by blocking caspase-3 and -9 in mitochondria.

However, an analysis of the activation level of microRNA-139 transfected into Tca8113 oral cancer cells by Ren et al. [108] indicated that miR-139 significantly inhibited cell proliferation and induced apoptosis. The pro-apoptotic effect of miR-139 molecules was associated with markedly decreased mitochondrial membrane potential, the induction of phosphatidylserine fusion, and activation of caspase-3, as well as activation of the Akt signalling pathway.

Other studies have found that *P. gingivalis* colonizing the oral mucosa protects oral epithelial cells from apoptosis by inhibiting the activation and binding of ATP to the purinergic receptor P2X7. Importantly, eATP released from stressed, dying, or infected cells binds to the P2X7 receptor and can enable pathogen elimination through several pathways: host cell death, inflammasome activation and IL-1β secretion, ROS and NO production or phospholipase D activation, promoting lysosome and phagosome fusion [109]. This is believed to be regulated by nucleoside diphosphate kinase (NDK), which promotes cell survival by preventing epithelial cell death. The main role of NDK is to catalyze the transfer of terminal phosphate groups from 5′-triphosphate- to 5′-diphosphate-nucleotides [109,110]. Importantly, inhibition of P2X7 receptor activity, by inducing ATP ligation and promoting heat shock protein (HSP-27) phosphorylation, affects both oral epithelial cells and the immunocompetent cells present in the oral cavity niche.

Aymeric et al. [111] report that cancer cells exposed to chemotherapy have the ability to produce extracellular ATP (eATP). The released eATP activates P2X7 receptors on dendritic cells (DC) stimulated by ionizing radiation and promotes the release of IFN-γ. Furthermore, signalling from the purinergic receptor activates the LRP3/ASC/caspase-1 inflammasome and stimulates IL-1β secretion. IL-1β cytokine production is needed to activate the anti-tumour immune response and polarize the CD8^+^ T lymphocytes that produce IFN-γ. As *P. gingivalis* infection reduces ATP production and activation on DCs, it impairs inflammasome functionality and inhibits IL-1 and IFN-γ production by cytotoxic lymphocytes. Similar information regarding the effect of NDK secretion by *P. gingivalis* on apoptosis and immune cell function was presented by Almeida-da-Silva [109]; *P. gingivalis* infection of human GECs was found to cause eATP hydrolysis. This phenomenon can inhibit ATP-induced apoptosis, reduce ATP-induced ROS and NO production via P2X7/NADPH oxidase signalling, and attenuate inflammasome activation, thus inhibiting the production and release of IL-1β.

Interestingly, Ohshima et al. [112] report that *P. gingivalis* can inhibit apoptosis phenomena through up-regulation of ZEB2 expression, a transcription factor which also controls epithelial–mesenchymal transition and inflammatory responses. The ZEB2 regulation by *P. gingivalis* was mediated through pathways involving β-catenin and FOXO1. Indeed, silencing ZEB2 induced cancer cell apoptosis and reduced their viability.

Importantly, all of the molecules and signalling pathways activated during *P. gingivalis* infection of the oral mucosa, with the aim of ensuring its survival, may constitute specific therapeutic targets in the treatment of periodontitis and oral cancer. Preventing host cell apoptosis may be a survival strategy for *P. gingivalis* in infected GECs. Therefore, further in-depth studies aimed at inhibiting the survival of *P. gingivalis* in GEC epithelial cells are needed to clarify the mechanisms facilitating colonization of the oral cavity niche.

**Figure 5:**
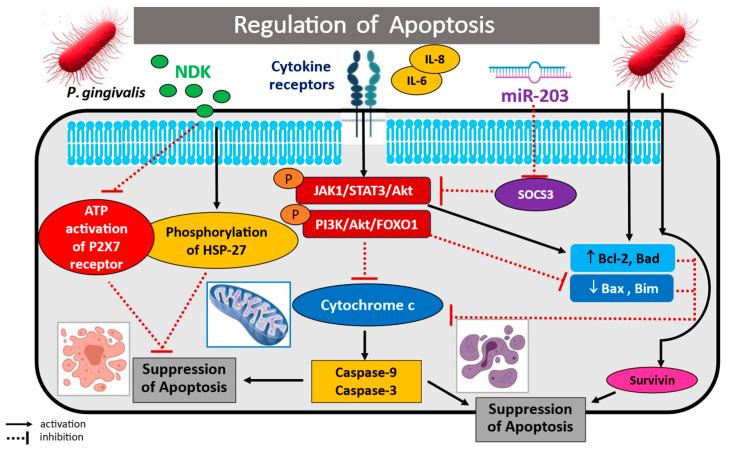
*P. gingivalis* inhibits intrinsic mitochondrial apoptosis through activation cytokine receptors and further activation of JAK1/STAT3/Akt and PI3K/Akt/FOXO1 signalling. This phenomenon inhibits cytochrome c release from mitochondria and further activation of caspase-e and caspase-9. Apoptosis can also be suppressed by *P. gingivalis*-induced overexpression of miR-203; this downregulates suppressor of cytokine signalling 3 (SOCS3), which in turn inhibits apoptosis through the activation of STAT3. NDK from *P. gingivalis* also inhibits ATP-dependent apoptosis through ligation of ATP with purinergic receptor P2X7. *P. gingivalis* enhances the expression of antiapoptotic molecules, i.e., Bcl-2 and Bad, and downregulates proapoptotic proteins, i.e., Bax and Bim.

The potential mechanisms of *Porphyromonas gingivalis* in regulation of apoptosis phenomena are illustrated in Figure 5.

The effects of *P. gingivalis* infection on oral epithelial and cancer cells in the selected in vitro models are shown in Table 1.

### 3.2. Animal Models of Oral Cancer

Recent years have seen the publication of a few papers describing experiments conducted in animal models of oral cancer, and discussing the key mechanisms of oral carcinogenesis associated with periodontal inflammation. Gallimidi et al. [76] examine the pathogenic role of chronic infection induced by *P. gingivalis* in cancer of the oral cavity in a mouse model of chronic periodontitis with oral carcinoma induced by 4-nitroquinoline-1-oxide (4NQO). After eight weeks of oncogene stimulation, increased signalling by the IL-6/STAT3 axis was noted, as well as direct interaction between the periodontal pathogen and oral epithelial cells via Toll-like receptors (TLRs). Morphometric and immunohistochemical analysis found that *P. gingivalis* chronic infection markedly enhanced the severity of the tongue tumours; STAT3 controls crucial genes driving proliferation, suppression of apoptosis, and aggressive tumour behaviour through the upregulation of cyclin D1. The tumours from the infected mice were 2.5 times larger, and significantly more invasive, than those from non-infected mice; the infected mice were also characterized by three-fold higher percentages of nuclear p65-positive epithelial cells than uninfected mice. The data confirmed that TLR2 plays a role in the induction of NF-κB transcription factor signalling. Importantly, the authors also propose another mechanism of oral carcinogenesis activation, in which bacterium-dependent OSCC cell proliferation is induced by the activity of key carcinogenic molecules such as cyclin D1, MMP-9, and heparanase.

Another animal model of oral carcinogenesis was also presented by Sztukowska et al. [77], who analyzed the ability of the oral pathogen to increase ZEB1 levels in carcinogenic model related to *P. gingivalis* infection. Gingival tissue was collected from BALB/c mice orally infected with *P. gingivalis* 33277 five times at two-day intervals (i.e., one, three, and eight days after the last infection with oral bacteria). It was found that colonization of oral tissues by *P. gingivalis* induced an increase in *ZEB1* mRNA expression in biopsy samples from OSCC within eight days compared to sham infected animals. The results confirmed that FimA-induced ZEB1 expression may be an important mechanism for inducing EMT phenomena and the subsequent development of OSCC.

Interesting observations were also obtained by Yao et al. [113] in animal models of OSCC in situ (CIS) associated with periodontitis-induced bacteria. Briefly, among eight-week-old Balb/c small mice, the diameter and mass of the primary tumour, and its formation and growth over three weeks, were higher in the study group colonized by *P. gingivalis* and *F. nucleatum* than in the control group. Furthermore, infection with oral pathogens led to extensive necrosis in the tumour tissue and numerous vascular lesions. The infected OSCC tissues also demonstrated a significantly higher proliferation index Ki67 and cyclin D1 expression. Further analysis of inflammatory cytokine expression in oral cancer revealed that periodontal pathogens promoted the expression of cytokines, i.e., IL-6, TNF-α, IL-18, apoptosis-associated granule protein containing CARD (ASC) (up to 6-fold), and caspase-1 (up to 4-fold), while significantly decreasing the level of transcription factor NF-κB, NOD protein, LRR, pyrin domain-containing protein 3 (NLRP3), and IL-1β (less than 0.5-fold). Furthermore, a significant increase in the number of CD4+ T lymphocytes, CD8+ T lymphocytes, and CD206+ macrophages was observed in the study group. Also, increased expression of γ-H2AX, p-ATR, RPA32, CHK1, and RAD51 were noted in tumour tissues, as well as a decrease in phosphorylation of the CHK1 signalling molecule (p-chk1).

Regarding the role of bacteria in oral mucosal biofilm, it has been found that *P. gingivalis* infection may promote distant metastases in the course of oral cancer and chemoresistance to therapeutic agents. Woo et al. [114] report that chronic and repeated infection of OSCC cells with *P. gingivalis* twice a week influences the immune response to chemotherapeutics and induces the ability to form metastases within the blood stream. The study also demonstrated that a xenograft mouse model composed of infected OSC-20 OSCC cells has higher resistance to Taxol, a well-characterized chemotherapeutic agent. The mechanism is believed to be associated with the activation of Notch1 (Notch intracellular domain 1, NICD). Furthermore, OSCC cells infected with *P. gingivalis* contributed to the formation of multifocal metastases in the lungs, unlike uninfected OSC-20 cells. Given the role of the Notch1 pathway in drug resistance in oral cancer, this signalling pathway may offer an important potential target for preventing chemoresistance to cancer therapy.

Unfortunately, despite the promising observations described above in mouse models of oral pathogen-induced carcinogenesis, only a few studies have analyzed the mechanisms underlying EMT, cancer development, and invasiveness of neoplastic lesions in these models. Therefore, further research is necessary to demonstrate the causal role of periodontal pathogens in OSCC.

#### Limitations of In Vitro and Animal Studies

To date, several preclinical studies have examined the role of *P. gingivalis* infection during subsequent stages of carcinogenesis and tumour progression in head and neck cancer. During the past two decades, several key in vitro and in vivo studies have been performed, as well as others carried out in tumour-bearing animals. These studies aimed to determine whether *P. gingivalis* is capable of modulating phenomena associated with carcinogenesis in oral cancer cells, such as apoptosis, differentiation, and proliferation.

Unfortunately, epidemiological data concerning the ability of *P. gingivalis* to regulate tumour initiation or modulate the course of cancer differs from preclinical data obtained in experimental models. This may be due to several reasons. Firstly, many studies lack methodological standardization and employ different laboratory methods. Secondly, many preclinical studies fail to consider as the quantity and functionality of *P. gingivalis* and its interactions with other oral biofilm pathogens, which can significantly influence its metabolic and anticancer role; they also often omit factors that induce in vitro oral dysbiosis, such as cigarette smoking and alcohol consumption, oral hygiene cultural behaviours, diet, and lifestyle. As such, it is not possible for preclinical researchers to unequivocally identify the relationships between metabolic competencies and pathways/biomarkers and the stages of carcinogenesis modulated by *P. gingivalis* with regard to clinical analyses and conclusions.

The effects of *P. gingivalis* infection on oral epithelial and cancer cells in the selected animal models are shown in Table 2.

### 3.3. Clinical Evidence in Human Samples

The oral microbiome is known to play a key role in the pathogenesis and development of periodontitis, inflammatory systemic diseases, and orodigestive cancers. Unfortunately, studies of human microbiome profiles have not yet provided a definitive answer to the role of different types of oral biofilm in the initiation and progression of periodontal bacteria-related diseases and orodigestive cancers. However, the development of new generation sequencing (NGS) technologies, i.e., 16S rRNA-based NGS gene sequencing, has identified many specific opportunistic species of human microbial communities that form a biofilm on the oral mucosa [115,116,117,118,119,121,122,123,124,125].

For example, Al-Hebshi et al. [115] compared the bacteriomes of squamous cell carcinoma biopsies and deep-epithelium swabs from matched control subjects, and examined the relationship between the bacterial membrane and OSCC. It was found that the periodontopathogen *Fusbacterium* spp. predominated in the inflammatory bacteriome, accompanied by inter alia *Streptococcus* spp., *Porphyromonas* spp., *P. gingivalis*, *Leptotrichia* spp., *Haemophilus* spp., *Prevotella* spp., *Campylobacter* spp., and *Neisseria* spp. Importantly, the abundance of each genus or species differed significantly between cases and controls. Additionally, the analysis showed that genes involved in bacterial motility, flagella formation, bacterial chemotaxis, and LPS synthesis were associated with a higher risk of carcinogenesis induction, whereas those responsible for DNA repair, purine metabolism, phenylalanine, tyrosine, and tryptophan biosynthesis, ribosome biogenesis, and glycolysis/gluconeogenesis were significantly more present in the control group.

Similar observations were made by Zhang et al. [116] in a study of OSCC patients using 16S rDNA sequencing. The authors propose that the development of oral diseases, systemic inflammatory illnesses, and oral squamous cell carcinoma may be associated with the disruption of the biological balance between biofilm bacteria and host. It was found that bacterial abundance and diversity was significantly higher in tumour-affected tissues in the buccal mucosa compared to healthy controls, and that these may represent potential diagnostic markers and therapeutic targets. The most abundant periodontal bacterial genus in the biofilm was *Fusobacterium* spp., but *Porphyromonas* strains were also confirmed to be involved.

Chang et al. present an interesting study of subgingival dental plaque and tumour tissues and peritumoural tissues from patients with OSCC using 16S rRNA amplicon sequencing, qPCR, and fluorescence in situ hybridization (FISH) [117]. The findings showed that periodontal pathogens were more abundant in cancer and paracancerous tissues than in subgingival plaque, with the highest abundance being found for *Porphyromonas gingivalis*, followed by *Fusobacterium nucleatum* and *Streptococcus sanguinis*. Using a special oligonucleotide probe, *P. gingivalis* was detected in 60.7% of OSCC biopsies, in 32.8% of paracancerous mucosa and only in 13.3% of normal tissues. Moreover, the obtained data showed that *P. gingivalis* infection was positively correlated with higher clinical stage, poor differentiation, and the presence of lymph node metastases in OSCC patients.

Another study took a metatranscriptome-based approach to analyzing the bacterial membrane in the oral cavity. Yost et al. [118] assessed the mRNA expression profile across the entire oral microbiome in OSCC with the aim of identifying specific molecular and metabolic functions associated with this disease. The carcinoid tissue biopsies and tumour-adjacent samples in cancer patients exhibited significantly higher numbers of transcripts of oral mucosa bacteria, including *Fusobacterium* and *Porphyromonas* strains, compared to location-matched oral sites from healthy subjects. The findings include specific metabolic signatures characteristic of OSCC.

In addition, the cancer samples and paraneoplastic tissues were found to demonstrate enhanced iron ion transport, tryptophanase activity, peptidase activity, glutamate dehydrogenase (GDH), starch synthase activity, and superoxide dismutase (SOD) compared to healthy buccal-matched sites. The neoplastic tissues also demonstrated enhanced microbiome processes and increased expression of virulence factors, such as capsule biosynthesis, flagellar biosynthesis, chemotaxis, cobalamin biosynthesis, iron transport, proteolysis, and hemolysin and adhesin activity [118].

Furthermore, the tissue from cancer patients exhibited enhanced protective activity against reactive nitrogen intermediates at non-tumour sites. As *P. gingivalis* is abundant in malignant oral epithelium, there is evidence to suggest it may be associated with gingival squamous cell carcinoma (GSCC). Indeed, an immunohistochemical (IHC) study of paraffin-embedded samples of squamous cell carcinoma and normal gingival tissues by Katz et al. [52] found *P. gingivalis* to be present in both normal gingival tissues and gingival cancer, with over 33% higher levels noted in neoplastic poorly differentiated gingival carcinoma specimens. Furthermore, *P. gingivalis*-specific IHC staining was at a two-fold higher level in cancer samples compared to control biopsies.

Li et al. [119] compared the composition and function of oral microbiota between gingival squamous cell carcinoma (GSCC) and chronic periodontal inflammation. The analysis included tissues from patients with periodontal cancer (GSCC), matched patients with periodontitis, and healthy individuals. Samples of saliva, subgingival plaque, tongue dorsum, buccal mucosa, neoplastic tissue, and paraneoplastic tissue were collected from each individual for taxonomic analysis using 16S rDNA amplicon sequencing and functional prediction. The analyzed periodontal pathogens comprised 46% of the bacteria in GSCC, 38.36% in subgingival plaque, and 44.13% in saliva. The predominant pathogens in neoplastic tissues were *Fusobacterium* and *Peptostreptococcus*, and to a lesser extent, *Porphyromonas* spp. Interestingly, GSCC was characterized by lower biofilm richness than periodontitis, where non-invasive bacteria associated with periodontal health predominated. Moreover, the subgingival plaque of GSCC was characterized by higher biosynthesis of lipopolysaccharides (LPS) produced by periodontal pathogens than the other tissues. Similarly, Perera et al. [121] also report lower abundance and species diversity of periodontal pathogens in neoplastic tissues in a case–control study of OSCC and fibroepithelial polyps (FEP); they also note a greater more abundance of pro-inflammatory bacterial features, including lipopolysaccharide biosynthesis and peptidases, in OSCC tissues.

#### 3.3.1. Translational Implications

Neuzillet et al. [122] examined the association between the presence of *Fusobacterium nucleatum* and *Phorphyromonas gingivalis* in tumour tissue and clinicopathological features, viz. disease recurrence and overall survival (OS), in two independent cohorts of patients; the first cohort had HNSCC from various primary sites, (oral cavity [OSCC], oropharynx, hypopharynx, and larynx), and the second consisted of OSCC patients. Importantly, tissue colonization by *F. nucleatum* correlated with older age, lower alcohol consumption, lower alcohol and tobacco consumption, and less frequent lymph node involvement. In addition, the multivariate analysis indicated that *F. nucleatum*-positive cases demonstrated lower disease recurrence rates and fewer metastases, as well as longer overall survival (OS), compared to infection-negative neoplasms. Gene and immunohistochemistry analyses related to immunotherapy showed that the Gram-negative bacterial load was inversely correlated with the M2 macrophages present in the tumour tissue.

An important, persistent clinical problem is chemotherapeutic resistance; this can affect fundamental treatments for highly aggressive human malignancies such as cisplatin, doxorubicin, 5-fluorouracil, gemcitabine, and tamoxifen. Oral cancer is also commonly associated with drug resistance and an uncontrolled invasive phenotype, which are the most common reasons for cancer recurrence and reduced survival. Recent data indicate that the Notch signalling pathway plays an important role in cancer development, invasive growth, and drug resistance. Importantly, after translocation to the nucleus, the Notch oncogenic molecule may dysregulate many key cellular pathways, including nuclear factor-kappa B (NF-κB), vascular growth factor receptor (VEGF), epidermal growth factor receptor (EGFR), platelet-derived growth factor (PDGF), mammalian target of rapamycin (mTOR), cyclin D1, *c-myc*, CDK p27^KIP1^, and p21^CIP1/WAF1^, Akt [123,124].

It has been noted that inhibiting the Notch pathway can increase drug sensitivity, leading to the inhibition of tumour growth, invasion, and metastasis [73,114,125]. For example, Ha et al. [73] analyzed the role of chronic *P. gingivalis* infection in the progression and drug resistance of oral cancer. To summarise, OSCC cells were infected with *P. gingivalis* twice weekly for five weeks. The cells acquired higher CD44 and CD133 antigen expression and a mesenchymal phenotype characterized by the expression of EMT markers. Long-term exposure to *P. gingivalis* also promoted the acquisition of migratory and invasive features associated with the generation of matrix metalloproteinases MMP-1 and MMP-10, which in turn was stimulated by the release of IL-8. Moreover, the described changes in the infected OSCC cells led to resistance to Taxol, a chemotherapeutic agent. Hence, inhibition of Notch signalling pathways may be a potential strategy for overcoming resistance to cancer treatment and may increase cisplatin (DDP) sensitivity when used in combination with conventional cytotoxic therapy.

Another important consideration associated with the treatment of oral cancer and other HNCs are its side effects, i.e., radiation-induced mucosal changes such as chronic necrotizing oral mucositis and xerostomia, as described in the phase III RTOG 97-09 randomized study [126]. The severity of changes in the exposed epithelium and gingival tissues has been found to increase with the presence of the oral commensal microbial communities in oral mucosa [126,127]. Both pathologies appear to develop as a result of chemotherapy and radiation therapy, leading to tissue inflammation, cell apoptosis or necrosis, and mucosal ulcerations induced by the resident oral microflora.

The most common periodontal pathogens in oral mucositis and xerostomia derive from the four bacterial genera *Fusobacterium*, *Treponema*, *Prevotella,* and *Porphyromonas*, including *P. gingivalis*, whose relative abundance varies during chemoradiotherapy. The changes occurring in response to CHRT-related therapy facilitate the colonization of the oral mucosa by various opportunistic and invasive bacterial species. Bacterial products and metabolic by-products may promote the production of inflammatory cytokines, and encourage cell proliferation, apoptosis, cellular invasion, and migration thorough host cell genomic alterations. They may also enhance macrophage activity to produce pro-inflammatory cytokines, thus sustaining chronic inflammation [120].

Xerostomia may also occur in response to reduced blood supply, impaired healing, and weakened host immune response. It can lead to changes in the composition of the gingival environment during irradiation of oropharyngeal tissues and contribute to the breakdown of mucus in the oral cavity, driving translocation of the pathogen into the lamina propria and the recruitment of inflammatory cells. As a result, anaerobic and microaerophilic bacteria are able to proliferate in the supra- or subgingival biofilm [128]. Further studies indicate the predominance of two genera, *Porphyromonas* and *Prevortella*, in the biofilm following chemoradiotherapy of oral mucositis. Interestingly, studies have shown lower amounts of *Porphyromonas* and *Prevotella* bacteria in the supragingival biofilm, while the numbers were significantly increased in the gingival sulcus in patients with gingivitis or chronic periodontitis. The presence of these microorganisms in the bacterial biofilm, directly related to poor oral hygiene, alcohol consumption, and tobacco smoking, induced adhesion and facilitated the colonization of the oral cavity by periodontopathogens after radiotherapy. Furthermore, significantly greater adhesion to neoplastic cells was noted in the presence of bacteria with fimbriae, which also supported the colonization of biofilm in irradiated individuals with xerostomia. Periodontal pathogens can also enhance the proteolytic activity of microorganisms on the fibronectin on the oral soft tissue surface via adhesion receptors; this promotes inflammation through the release of pro-inflammatory cytokines IL-1 and IL-6 during radiotherapy-induced mucositis [129]. It is important to emphasize that unfortunately, only symptomatic treatment is currently available for patients suffering from radiation-induced xerostomia. Therefore, while antibacterial therapy could offer significant therapeutic potential in the treatment of xerostomia, large-scale randomized human trials are needed to confirm its effectiveness in clinical settings.

#### 3.3.2. Limitations of Clinical Trials

It is worth noting that the clinical studies conducted to date are beset by a number of characteristics that make comparison difficult, such as small group sizes, heterogeneity of patient samples (biopsy material from lesions or normal oral epithelium, saliva), and insufficient sample size, which may cause errors and potential systematic bias, thus hindering comparative analysis between patients and control groups; also, different observation periods, stimulation methods, and bacterial infection rates, or varying numbers of repeated experiments, may also contribute to discrepancies in the final conclusions

In addition, many researchers fail to consider the variability in the activity and function of the periodontal pathogens, which can be influenced by cultural behaviours, alcohol consumption, tobacco smoking, body mass index, and socioeconomic status, as well as oral hygiene in patients with periodontitis and oral cancer. Furthermore, study populations may consist of heterogeneous ethnic groups, with different cultural behaviours, diets, and lifestyles.

Furthermore, many studies are based on heterogeneous clinical data, which also prevents in-depth comparative analysis or precise stratification and sensitivity analyses. Studies may use different species of bacteria, and vary in the quantitative exposure to bacterial infection, and the repeatability of the experiments. The activity of the periodontopathogens may also be measured in different laboratories at different times, using different research methods, which also limits the generalizability of the final results. Importantly, clinical studies investigating the coexistence of *P. gingivalis*, *F. nucleatum*, and other cooperating bacteria in oral biofilms in cancer patients are primarily cohort studies, which are burdened by a low level of evidence. Therefore, a systematic review based on randomized controlled trials is necessary to confirm the association between the coexistence of different bacterial species, their interplay (i.e., cooperation or antagonism), and cancer development.

The effects of *P. gingivalis* infection on oral epithelial and cancer cells in the selected clinical studies are shown in Table 2.

### 3.4. Community Interaction of Periodontal Pathogens in Oral Carcinogenesis

The results of epidemiological studies conducted over the past few years indicate a complex relationship between the diverse, multi-species bacterial community inhabiting the oral environment, periodontal inflammation, and the occurrence of oral cancer. The bacteria of the oral biofilm constitute a diverse and complex community of microorganisms. Within the tight biofilm matrix, periodontal pathogens communicate with each other and exchange nutrients, competing or cooperating; as such, they act as an interdependent pathogenic unit with a distinct division of functions [130].

The best-known and most abundant periodontal pathogens in the human oral microbiome are *Porphyromonas gingivalis* and *Fusobacteria nucleatum*, which together influence the regulatory mechanisms responsible for chronic periodontal inflammation, infection-related systemic diseases and oral cancer. The interaction between *P. gingivalis* and *F. nucleatu* has been the subject of a number of clinical and experimental studies; unfortunately, there is still insufficient and unambiguous evidence regarding the synergy between them [131]. It is undisputed that the diverse bacterial species occupying the human oral biofilm compete for environmental niches and play a crucial role in maintaining balance and homeostasis in the complex ecosystem of the oral cavity. In recent years, increasing attention has been focused on the role of the oral microbiome and its influence on the development of bacteria-dependent systematic disease, chronic periodontal status, and, most importantly, the development of cancers, including oral cancer [132,133,134].

Progress in next-generation sequencing (NGS) technology and bioinformatics tools over the last decade has allowed large communities of microorganisms to be studied as a whole, rather than as a few individuals. Disturbances in the quantitative and qualitative relationships between periodontal pathogen species can lead to dysbiosis and the disruption of the host immune response; they can also contribute to the development of serious diseases, such as dental caries and periodontitis, cardiovascular diseases, rheumatoid arthritis, Alzheimer’s disease, lung diseases, and cancer [135,136]. Recent discoveries have shown that under pathological conditions, complex signalling interactions occur between the cellular and non-cellular factors present in the extracellular matrix of the oral biofilm, including components of the bacterial microbiome. Importantly, these phenomena may directly induce metaplasia and carcinogenesis, increase local and distant invasiveness, and encourage resistance to chemotherapeutic agents and immunotherapy [137,138].

Both oral squamous cell carcinoma (OSCC) and gastrointestinal cancers have been found to be associated with the oral microbiome [139]. However, the overwhelming majority of studies in the area have focused on the role of a specific single bacterial species, despite the fact that tumour tissue harbours a broad population of microorganisms. Among these, *P. gingivalis* is believed to easily associate with other bacterial species, such as *Fusobacterium nucleatum*, *Treponema denticola*, and *Prevotella intermedia*. For example, the fimbriae of *P. gingivalis* are believed to increase coaggregation with other bacteria species, such as *Streptococcus gordonii*, *Veillonella* spp., and *Aggregatibacter actinomycetemcomitans*. Moreover, in patients with periodontitis, such associations may stimulate the invasion of human gingival epithelial cells and even influence the transmission of *P. gingivalis* to other distant body sites [140,141,142].

One of the best-known mechanisms of mutual cooperation in oral cancer is the coexistence and interdependence of *P. gingivalis* and *F. nucleatum*, although the exact role and potential impact of this interaction on carcinogenesis remain unclear [143,144,145,146]. These two pathogens have been found to co-occur in oral biofilms in infectious oral diseases such as periodontitis, and in oral squamous cell carcinoma (OSCC) [116,117,147,148]. For example, a cross-sectional study by Zhang et al. [116] confirmed the coexistence of *P. gingivalis* and *F. nucletum* in the buccal mucosa of patients with OSCC using 16S rDNA sequencing. It was found that bacterial diversity and abundance were significantly higher in the tumour tissues compared to the normal buccal tissues. Furthermore, the analysis confirmed that genes involved in bacterial chemotaxis, flagellar formation, and lipopolysaccharide (LPS) biosynthesis were also significantly upregulated in the OSCC group. The researchers also propose that the coexistence and interaction of *P. gingivalis* and *F. nucleatum* may represent diagnostic markers and potential treatment targets [116].

Similar conclusions were also reached by Torralba et al. [147], who identified significantly higher levels of both *P. gingivalis* and *F. nucleatum* in OSCC tissue samples compared to samples taken from healthy individuals. The microbial composition and functional activity of oral biofilms were compared with virulence factor levels using 16S rDNA sequencing and metagenomic analysis in oral squamous cell tissue, non-tumour tissue, and saliva from patients with OSCC.

Similarly, Chang et al. [117] examined tumour tissues, peritumoural tissues, subgingival plaque samples, and a group of normal tissues using qPCR. The authors also noted that both *P. gingivalis* and *F. nucleatum* were present in higher concentrations in tumour tissues than in normal tissues, confirming previous observations. Interestingly, the relative abundance of these two bacterial species in tumour tissues was positively correlated with the amount of bacteria in subgingival plaque, confirming the link between periodontitis and oral cancer. Furthermore, compared to *F. nucleatum* infection, *P. gingivalis* infection was significantly more strongly associated with late clinical cancer stage, poorly differentiation of tumours, and the presence of nodal metastases in patients with OSCC.

Similar observations were also reported by Park et al. [148] in a cohort study of patients with *P. gingivalis* infection. Higher levels of serum IgG and IL-6 determined by ELISA were found to be directly associated with worse OSCC prognosis, even though IgGs to both periodontopathogens were confirmed in the OSCC cohort. The researchers propose that these serum IgG antibodies, and serum IL-6 in cases of OSCC, may have diagnostic potential and serve as biomarkers for differentiating these patients from healthy controls, and for preparing a prognosis for OSCC.

In summary, clinical evidence clearly showed that the coexistence of *P. gingivalis* and *F. nucleatum* in OSCC was associated with a poorer prognosis. However, while bacterial species other than *P. gingivalis* and *F. nucleatum* may also influence and modulate the complex functions of the cancer-associated microbiome, further in-depth studies are necessary to confirm their role.

Several studies using in vitro cultures and animal models have confirmed that *P. gingivalis*/*F. nucleatum* co-infection has a potentially greater ability to promote oral cancer carcinogenesis than either species acting alone in the mucosal biofilm. This cooperation involves inter alia the activation of Toll-like receptors present on the plasma membrane of oral epithelial cells, leading to increased production of pro-inflammatory molecules and cytokines, enhanced EMT, increased expression of genes associated with inflammation, migration, invasion and cell cycling, and the inhibition of cell apoptosis [113,149]. However, further evidence is needed to unequivocally demonstrate the synergy of the *P. gingivalis*/*F. nucleatum* system in contributing to carcinogenesis.

Despite these limitations, a few studies indicated that the mechanisms promoting increased tumourigenesis in OSCC in the case of *P. gingivalis*/*F. nucleatum* coexistence are the activation of key molecules for tumour mass growth and unrestricted proliferation of oral epithelial cells, such as cyclin D1, and proangiogenic and pro-inflammatory cytokines such as IL-6 and IL-8, TNF-α, IL-18, apoptosis-associated granule protein containing CARD (ASC), MMP-9, and caspase-1. This process also lowers the expression of nuclear factor NF-κB, NOD protein, LRR, and NLRP3, as well as IL-1β. Furthermore, in the studied systems, the *P. gingivalis*/*F. nucleatum* group significantly increased the number and activity of CD4+ T lymphocytes, CD8+ T lymphocytes, and CD206+ macrophages [113,149].

Importantly, many analyses do not include a control group for both periodontal pathogens. Therefore, it is difficult to conclude that the final study results truly indicate synergy in the action of both biofilm pathogens in the oral cavity. Only two studies have compared the effects of these two species with their corresponding single species [75,77]. Sztukowska et al. [77] found that infection with *P. gingivalis* alone enhanced the activity of the transcription factor FimA-driven ZEB1 and promoted the migration of infected gingival epithelial cells (TIGKs) in vitro. The induction of ZEB1 expression by *P. gingivalis* was further increased by the coexistence of *F. nucleatum* with *P. gingivalis*. The researchers conclude that it is necessary to consider the synergy or competition between *P. gingivalis*/*F. nucleatum* when examining carcinogenesis. Similar results were presented by Lee et al. [75], who also observed that infection of the oral epithelium with *P. gingivalis* alone increased the expression of key transcription factors promoting EMT, including ZEB1. They also note, however, that the combined action of these opportunistic pathogens slightly enhanced cell migration compared to either bacterial species alone. Interestingly, Ohshima et al. [112] also indicate that *P. gingivalis* activated ZEB2 induction and β-catenin activation. *P. gingivalis* controlled ZEB2 function via dephosphorylation of the serine 256 and serine 329 residues in FOXO1.

Interestingly, another mechanism of *P. gingivalis* and *S. gordonii* cooperation has been proposed. Fitzsimonds et al. [150] report that *P. gingivalis* and its gingipain proteases are regulated by olfactomedin 4 (OLFM4), which may serve as a potential biomarker of EMT in head and neck squamous cell carcinoma. *P. gingivalis* gingipain proteases control OLFM4 function by activating the Notch1/Jagged1 signalling cascade and cleaving the extracellular domain of Notch1. Interestingly, *P. gingivalis* gingipains are stimulated by *S. gordonii* with hydrogen peroxide, thereby antagonizing Notch signalling [150]. Similarly, Marimuthu et al. [151] confirm the higher abundance of some markers in the secretome derived from HNSCC tumour cells, including olfactomedin-4, OLFM4, hepatocyte growth factor activator, HGFA, insulin-like growth factor binding protein 3, IGFBP3, and opioid growth factor receptor, OGFR, reaching 70–75% of the studied cases.

Moreover, Periasamy et al. [141] propose that co-infection with *P. gingivalis* and *F. nucleatum* may also enhance cancer development due to the differences in nutrient utilization by the two species and their different functional pathways. Specifically, *P. gingivalis*, due to its proteolytic nature, primarily degrades dipeptides, while *Prevotella* and *F. nucleatum* degrade smaller molecules and amino acids. Therefore, this suggests metabolic synergy, with *P. gingivalis* providing amino acids essential for the functioning of *F. nucleatum*, which lacks proteolytic capabilities. This mutualistic interaction favours co-colonization of the coexisting bacteria.

Perera et al. [121] identified a significant factor influencing the functional coexistence of periodontal bacteria during oral tissue inflammation and its influence on carcinogenesis in OSCC. The researchers emphasize the importance of considering the functional redundancy of oral bacteria; as such, studies should not only focus on biofilm composition, but also aim to predict the functional roles of periodontal pathogens and the mechanisms activated by them in OSCC. It is hence worth noting that *P. gingivalis* promoted the development of oral cancer through various mechanisms, including immune evasion, inhibition of apoptosis, and enhanced EMT; it also acted functionally, through enhanced pro-inflammatory activity, including lipopolysaccharide and peptidase biosynthesis. In contrast, *F. nucleatum* promoted cancer development through proliferation, immunosuppression, and promotion of metastasis. Therefore, it is possible that co-infection by *P. gingivalis* and *F. nucleatum* may enhance tumourigenesis and subsequent tumour development to a greater degree than infection with a single species.

So, to summarise, tumourigenesis and development and further cancer progression does not depend directly on the abundance of individual bacterial species but may be linked to the functioning of the entire microbial community, consisting of hundreds of bacterial species. Hypotheses that describe the relationships between different bacterial species in the biofilm can be introduced in two basic models: the “driver–passenger” model and the cancer–microbiome–immunity axis for colorectal cancer [152,153]. These hypotheses proposed that pathogenic bacteria can initiate cancer development and function as a “driver”. The driver-induced changes in the tumour microenvironment and cellular metabolism lead to a competitive advantage for parasitic bacteria, which are opportunistic “passenger” pathogens, which subsequently either suppress or promote cancer progression. Phenomena related to this theory in oral cancer carcinogenesis were explored by Al-Hebshi et al. [154]. The authors presented a modified, new, in vitro functional “passenger–tuning–driver” model of the oral cancer microbiome, with particular emphasis on the carcinogenicity of periodontal bacteria, *P. gingivalis* and *F. nucleatum*, based on available clinical trial results. Contrary to the “driver–passenger” model, it was concluded that periodontal pathogens of the oral microbiome were not involved in the initiation of OSCC. The researchers emphasized that the results obtained in this area are inconsistent, and the composition of the microbiome associated with oral cancer varies significantly. The discrepancies in the final conclusions could be due to the methodological differences of the studies, but also to the phenomenon of functional redundancy. This phenomenon occurs when different species of oral biofilm, constituting a “passenger”, may be enriched with “driving factors” in different samples but still perform the same functions. The impaired function of the initially “passenger” microbiome in the tumour microenvironment became a functional “driver” as the intratumoural microbiome matured through the expression of pro-inflammatory components and virulence factors, thus contributing to oral cancer progression. The second functional concept of the cancer–microbiome–immunity axis, proposed by Jain et al. [153], describes the interplay between the microbiome, immunity, and cancer. In this model, periodontal pathogens can cooperate with cancer cells by inducing them as antigens/proto-oncogenes or contribute to the generation of indirect adjuvant signals that lead to immunomodulation. These signals, produced and secreted by cancer cells and influencing their function, can include metabolites, toxins, and vesicles, or cytokines secreted as a result of modulating the tumour–host cell relationship.

Other studies examine the coexistence of the periodontal co-pathogens *P. gingivalis* and *F. nucleatum* in chronic inflammatory conditions of the oral and gingival tissues, i.e., periodontitis. Polak et al. [155] compare the effect of infection by the two pathogens individually and together in mouse periodontal tissues. The findings indicate that *P. gingivalis*/*F. nucleatum* co-infection was characterized by increased alveolar bone loss and TNF-α and IL-1β levels compared to infection by individual agents. The researchers emphasize that polymicrobial infection with *P. gingivalis*/*F. nucleatum* exacerbates bone scaffold destruction and induces a more severe inflammatory response than for individual infection. Similar findings were reported by Maekawa et al. [156], who demonstrated that *P. gingivalis* can manipulate crosstalk between TLR2 receptor and the complement receptor C5aR, thus uncoupling bacterial clearance from inflammation and promoting dysbiosis. Interestingly, the findings indicate that neutrophils protect bacteria by inhibiting the host-protective TLR2-MyD88 pathway through proteasomal degradation of MyD88, while simultaneously enhancing the alternative TLR2-Mal-PI3K pathway, thus inhibiting phagocytosis.

Similar interesting observations were reported by Saito et al. [157], who confirmed that *F. nucleatum* can enhance the invasion of *P. gingivalis* into the Ca9-22 immortalized human gingival cell line; the cells were doubly infected with *P. gingivalis* ATCC 33277 and *F. nucleatum* TDC 100. It was found that both periodontal pathogens formed consortia, and the presence of *F. nucleatum* facilitated the penetration of *P. gingivalis* into the cells. Furthermore, the coexistence of *P. gingivalis*/*F. nucleatum* was found to colocalize with a lipid raft marker, GM1-containing membrane microdomains; for each species, the duration of infection increased host cell invasion by *P. gingivalis*, including its serine phosphatase mutant SerB, and complementary strains.

Periasamy et al. [141] report that *P. gingivalis* ATCC 33277 exhibits a significant ability to interact with various early, mid-, and late colonizers of oral biofilm, which grow exclusively in saliva. In a study of biofilm communities, *P. gingivalis* was unable to grow as a single species even with primary oral colonizers *Streptococcus oralis*, but it did exhibit mutualistic growth in association with two other primary colonizers, *Streptococcus gordonii* and *Actinomyces oris*, as well as with *Veillonella* spp. (an early colonizer), *Fusobacterium nucleatum* (mid-colonizer), and *Aggregatibacter actinomycetemcomitans* (late colonizer). Hence, *P. gingivalis* can be present at all stages of dental plaque development, regardless of the co-presence of other oral commensal bacteria, thus promoting the development of periodontitis.

In summary, both *P. gingivalis* and *F. nucleatum* are believed to initiate the development of cancer and promote its subsequent invasiveness via a range of mechanisms; this poses a serious challenge for the development of targeted therapies, as effective therapy must address a number of targets. It is also possible to create effective cancer therapies based on microbial modulation by exploiting the “driver–passenger”/”passenger–tuning–driver” models and theories of the cancer–microbiome–immunity axis; it is theoretically possible to increase the effectiveness of immunotherapy and reduce its toxicity by modulating the microbiome, which forms biofilms and influences dysbiosis. For example, studies have examined the effect of introducing antibiotics, probiotics, and prebiotics to modulate the microbiome in anticancer treatments [158,159]. However, further testing is needed to confirm the clinical effectiveness of these approaches as potential cancer treatments.

### 3.5. Summary of Limitations of Studies on P. gingivalis for Oral Carcinogenesis

When studying the role of periodontal pathogens such as *Porphyromonas gingivalis* in oral carcinogenesis and disease progression, which act by remodelling the tumour microenvironment, it is important to note the limitations of both in vitro research, animal models, and clinical trials. Most importantly, since the introduction of next-generation sequencing technology (NGS) such as 16S rRNA metagenomics, the majority of publications have focussed on the role of specific, individual bacteria in the oral biofilm, without considering the presence of other pathogens in the tumour niche microenvironment. Indeed, most publications ignore the community nature of periodontal bacteria and fail to consider that the abundant bacteria together form a unique pathogenic unit.

Indeed, to draw true and reliable conclusions from in vitro and in vivo research on the role of the periodontopathogen *P. gingivalis*, the human oral biofilm should be regarded as a collection of diverse, opportunistic bacterial species constituting a unique ecosystem, cooperating or competing with each other in the oral microenvironment. A biofilm enables the formation of a specific complex pathogenic microbial community. In the oral mucosa, it facilitates effective communication and nutrient exchange between pathogens, and the activation or inhibition of intracellular signalling pathways needed for survival. The community can also exert pathogenic effects; for example, disturbed relationships between periodontal pathogen species can induce dysbiosis and disrupt host immune response.

Unfortunately, the potential role of the oral microbiome in the development of bacterial-dependent systemic diseases and periodontitis, and the initiation of tumours such as oral cancer, is poorly understood. This may be attributed to the predominant research model, which focuses on the activity and function of only a single bacterial species present in the complex ecosystem of the oral cavity. Therefore, future research programmes should include the complex synergistic/competitive signalling interactions between the various bacteria inhabiting the oral cavity, host cells, and immunocompetent cells present in the extracellular matrix; a broader perspective such as this can identify potential signalling pathways, specific molecules and virulence factors, and define their importance in the etiology of bacteria-related oral and systemic diseases, as well as cancer development. Furthermore, oral microbiome research frequently neglects the variability in the activity and function of periodontal pathogens, and the influence of lifestyle factors such as alcohol consumption, tobacco smoking, and oral hygiene, as well as body mass index (BMI) and socioeconomic status.

Another important limitation of research on the role of *P. gingivalis* in oral cancer is that the publications tend to focus solely on analyzing the composition of the microbiota, and not the functional redundancy among oral bacteria. Unfortunately, to date, no attempts have been made to perform any in-depth or definitive functional analysis of the bacteriome community in relation to oral cancer. As such, further extensive analyses to explore the actual association between the microbiota at functional levels are needed.

In addition, significant methodological differences can be seen between studies, with some using microarray approaches such as 16S rRNA metagenomics, microbial profiling, bioinformatics, and -omics technologies, and others using more established ELISA and IHC methods. Modern microarray technologies should also be standardized with regard to DNA isolation, the selection of primers specific for amplification of hypervariable regions, and bioinformatic methods for microbiota analyses. Sample collection should also be standardized, i.e., biopsy, surface swab or saliva, as should the selection of control groups, i.e., patient control material from a specific individual or healthy control persons.

## 4. Conclusions

This narrative review summarizes the current state of knowledge regarding the role of *Porphyromonas gingivalis*, a keystone pathogenic bacterium in human oral microbiota, and its involvement in the community action of bacteria present in the bacterial film in the oral cavity. The work also analyses the mechanisms and signalling pathways that play a part in maintaining the proper morphology and function of oral tissues; it also describes the involvement of *P. gingivalis* and other cooperating/counteracting pathogens such as *F. nucleatum* in periodontitis and the initiation of carcinogenesis. These pathogens are known to influence the activity of epithelial-to-mesenchymal transition and tumour spread, pro-inflammatory effects, host immune response, cell cycling and proliferation, and apoptosis. By synthesizing numerous in vitro and in vivo studies, mostly from the last two decades, the review offers a clearer understanding of the carcinogenic potential of *P. gingivalis* and the key mechanisms of its action on oral cancer, the most common head and neck cancer. It provides a detailed assessment of the analyzed biological material with regard to cohort types, diagnostic methods, and the role of virulence factors, such as FimA and gingipains. It thus clarifies the potential role of *P. gingivalis* in the initiation, development, and metastasis of OSCC tumour cells. It also indicates important regulatory signalling pathways for tumourigenesis, the influence of periodontal pathogens on the function of host immune cells, and the potential of intracellular checkpoints and induced tumour antigens.

Three key conclusions emerge from this narrative review:

(1) Importantly, the molecules regulating the functions of *P. gingivalis* and its relationship with other oral biofilm pathogens may represent potential therapeutic targets for preventing oral cancer and inhibiting its development. They may also constitute an important potential element of multitargeted therapeutic strategies to block biofilm formation or disrupt the established bacterial membrane matrix in the oral cavity. The bacterial-dependent pathology of oral cancer therefore represents a rapidly evolving field of oncology, although it requires a precise understanding of oral mucosal biofilm formation and function, the natural antibacterial defence against periodontal pathogens, and the role of the bacterial community in supporting the cancer cell escape from immune surveillance.

(2) Through the development of modern cytotoxic or combination therapy, and nanotechnology-based drug delivery systems that improve the penetration of anti-tumour agents and factors into the oral biofilm, modern pharmacology can improve the efficacy of existing treatment and offer personalized antibacterial therapy. This, however, requires a thorough understanding of influence of the complex pro- and anti-tumour pathways activated during infection by pathogens such as *P. gingivalis*, as well as the interactions between the microbial community complex and tumour and immunocompetent cells. It is also crucial to more accurately identify the population of oral cancer patients who could most benefit from the therapy. Nevertheless, in vitro studies, clinical trials, and mouse models of periodontitis/oncogenic-related carcinogenesis confirm that chronic bacterial infection may potentially promote the development of OSCC through direct interaction of periodontal pathogens with neoplastic and precancerous oral epithelial cells via Toll-like receptors (TLRs) P2 × 7, C5aR, or B7H1 receptors.

(3) It is also increasingly emphasized that the interaction of *P. gingivalis* with commensal microbiota, such as *F. nucleatum*, and recognized risk factors such as alcohol consumption, cigarette smoking, and poor oral hygiene, may play a significant role in increasing the risk of developing oral cancer and worsening patient prognosis. It has been suggested that these factors may act synergistically in the pathogenesis of OSCC. Studies aimed at identifying multiple mechanisms of polymicrobial synergy or antagonism in oral cancer, utilizing serum IgG antibodies against *P. gingivalis*, and determining the role of key biochemical and metabolic factors associated with infection may contribute to improved diagnosis, personalized therapy, and prognosis for oral cancer.

These goals can best be achieved by molecular analyses using modern metagenomic methods and nanotechnology, as well as in-depth epidemiological and mechanistic studies using larger cohorts and better control. Furthermore, future studies on the role of bacterial consortia in multiple disease processes may reveal new complex relationships between microbiomes and cancer cells. Therefore, there is a need for more extensive and accurate in vitro and in vivo research and clinical trials aimed at obtaining reliable data; these findings can provide a deeper understanding of the microbial interplay that can support the development of innovative early diagnosis and treatment strategies in oral cancer.

## Figures and Tables

**Table 1:** The effect of *P. gingivalis* infection on oral epithelial/cancer cells in the selected in vitro models.

Author	*P. gingivalis* in In Vitro Models of OSCC	
	Study Design and Methodology	Key Findings
In vitro studies		
Ha et al. [73]	- The human oral squamous cell carcinoma (OSCC) cells infected with *P. gingivalis* twice a week for 5 weeks - SCC-25, OSC-20, and SAS human OSCC cell lines - Real-time quantitative PCR (qRT-PCR) analysis	- *P. gingivalis* ⟶ morphological changes in host cancer cells into an elongated shape (mesenchymal phenotype) vs. the control - ↑ CD44 and CD133 - ↑ invasiveness of *P. gingivalis*-infected OSCC cells - ↑ tumour aggressiveness ⟶ ↑ MMP-1 and MMP-10 and EMT phenomena - ↑ MMP-1 and MMP-10 stimulated by IL-8 release
Abdulkareem et al. [35]	- The OSCC H400 human cell line - Cultures treated with the heat-killed *P. gingivalis* - The control group treated with media only - Semi-quantitative reverse transcriptase-polymerase chain reaction (sq-RT-PCR) and PCR-array analysis - Immunofluorescence and IHC analysis	- Periodontal bacteria modulate vimentin and E-cadherin expression in H400 cells after 8 days of culture - ↑ vimentin vs. the control - ↓ E-cadherin vs. the control - H400 cells exhibit mesenchymal-like morphology after *P. gingivalis* infection
Gallimidi et al. [74]	- SCC-25 and CAL-27 human squamous tongue SCC cell lines (TSCC) - Cells were incubated multiplicity of infection (MOI) with *P. gingivalis* MOI 100 and/or *F. nucleatum* MOI 5 - Real-time quantitative PCR (qRT-PCR) analysis	- Exposure to each of the pathogens or to a mixture of both ⟶ ↑ IL-6 by SCC-25 and CAL-27 cell lines, ↑ cytokines, enzymes and bioactive molecules implicated in oral cavity SCC proliferation, survival and aggressiveness, i.e., cyclin D1, TNFα, MMP9, and heparinase - Inhibition of TLR2 with neutralizing antibody abrogated the effect of *P. gingivalis*/*F. nucleatum* on SCC-25 proliferation
Cho et al. [75]	- The human oral squamous cell carcinoma (OSCC) cell line YD10B - Non-infected controls - YD10B cells were cocultured with live *P. gingivalis* strain 381 - 16S rRNA, Flow cytometry analysis, Western blot analysis, Multiplex bead assay, and Enzyme-linked immunoassay (ELISA)	- *P. gingivalis* ⟶ morphologic changes in YD10B OSCC cells, such as the loss of adhesiveness and a polygonal shape vs. the control - ↑ invasiveness of *P. gingivalis*-infected YD10B cells - ↑ CD44 and CD133 - ↑ α-SMA and vimentin, Slug and Twist - ↓ cytokeratin 13 - ↑ MMP-1, 2, 9, and 10 vs. non-infected controls - *P.* gingivalis ⟶ ↑ IL-8
Lee et al. [76]	- Primary human oral epithelial cells (OECs) - Non-infected OECs controls - *P. gingivalis* ATCC 33277 was cultured (MOI 100) - Western blot analysis, scratch migration assay, immunofluorescence microscopy, Sybr green quantitative RT-PCR	- *P. gingivalis* ⟶ ↑ phosphorylated GSK3β vs. uninfected OECs - *P. gingivalis*-infected OECs ⟶ ↑ Snail, Slug and ZEB1 EMT-inducing transcription factors vs. uninfected OECs - ↓ E-cadherin vs. the control - ↑ β-catenin vs. the control - ↑ MMP-2, 7, and 9 vs. non-infected controls - ↑ vimentin vs. the control
Sztukowska et al. [77]	- *P. gingivalis* ATCC 33277, *F. nucleatum* ATCC 25586 strains - Human telomerase immortalized keratinocytes (TIGKs) derived from gingival epithelium - Immunoblotting, quantitative reverse transcription-PCR (qRT-PCR), luciferase reporter assay, immunofluorescence, and immunohistochemical staining	- *P. gingivalis* ⟶ ↑ ZEB1 in TIGK cells in a FimA-dependent manner - ↓ miR-200 ⟶ ↑ level of ZEB1 mRNA - ↓ miR-200b, miR-200c and miR-205 levels - ↑ N-cadherin, vimentin and matrix metalloproteinase MMP-9 - *P. gingivalis* ⟶ ↑ the migration of TIGK cells into matrigel
Yao et al. [78]	- *P. gingivalis* ATCC 33277 strain - Human primary cultures of gingival epithelial cells (GECs) - Annexin-V and propidium iodide staining, RNA interference, Western immunoblotting, immunoprecipitation, Real-time quantitative polymerase chain reaction (qRT-PCR)	- ↓ Akt by siRNA reverses *P. gingivalis*-induced protection of GECs against cell death - Akt signalling ⟶ ↑pro-apoptotic Bad phosphorylation in GECs infected by *P. gingivalis* - *P. gingivalis* ⟶ ↓ Bax and ↑ Bcl-2 - *P. gingivalis* ⟶ ↓ caspase-3, but did not have any effect on the caspase-9 activation
Hoppe et al. [79]	- Immortalized oral keratinocytes (OKF6) induced by the keystone pathogen *P. gingivalis* - Real-time polymerase chain reaction (RT-PCR), enzyme-linked immunosorbent assay, Western blot, and protein arrays, trypan blue staining, matrigel assays, and anoikis-assays	- *P. gingivalis* ⟶ ↑ proliferation rates, ↑ PI3K/Akt signalling and the mTOR-pathway - Inhibition of GSK3β ⟶ ↑ β-catenin and Snail, ↓ E-cadherin to N-cadherin - *P. gingivalis* ⟶ ↑ Oct3/4, Sox2, and Nanog
Liu et al. [80]	- Human telomerase immortalized gingival keratinocytes (TIGK) and OKF6/TERT cells and EC9706, SCC9 and HeLa cells - *P*. *gingivalis* WT, Δ*ltp1*, or Δ*php1* mutants - Tyrosine phosphatases *ltp1* and *php1* were amplified by PCR - Quantitative reverse transcription PCR (qRT-PCR), immunofluorescence, immunoblotting, enzyme-linked immunosorbent assay, phospho-antibody array	- Ltp1 secreted within gingival epithelial cells - An *ltp1* mutant of *P*. *gingivalis* ⟶ ↓ ability to induce epithelial cell migration and proliferation - Ltp1 ⟶ ↑ the transcriptional upregulation of Regulator of Growth and Cell Cycle (RGCC) - *P. gingivalis* ⟶ ↑ RGCC expression through Akt, activated by phosphorylation on S473 and ↓ PTEN in epithelial cells - Knockdown of RGCC ⟶ ↓ ZEB2 and IL-6 production
Utispan et al. [81]	- Human primary (HN18, HN30, and HN4) and metastatic (HN17, HN31, and HN12) HNSCC cell lines - Human monocyte THP-1 cells differentiated into macrophages incubated with *P. gingivalis* LPS - Real-time PCR (RT-PCR) and ELISA	- The LPS-induced macrophages ⟶ ↑ IL-6 and CD14 expression - The *P. gingivalis* LPS ⟶ ↑ macrophage NO secretion, and ↓ TNF-α production - The LPS-induced macrophages ⟶ ↓ HN4 proliferation and invasion of all HNSCC cell lines
Groeger et al. [82]	- *P. gingivalis* strain W83 - The human squamous cell carcinoma cell line SCC-25 and primary human gingival keratinocytes (PHGK) - Human Antibacterial Response RT^2^ Profiler array, quantitative reverse transcription PCR (qRT-PCR)	- SCC-25 cells treated with *P. gingivalis* W83 ⟶ ↑ TLR signalling cascade, NF-κB pathway and the MAPK pathway genes, i.e., *IRAK1*, *IRAK3*, *IRF5*, *TICAM1*, *TRAF6*, *IKBKB*, *NFKB1*, *RELA*, *Jun*, *MAP2K1*, *MAP2K4*, *MAPK1*, *MAPK14*, *MAPK8*, and the cytokine IL-*12* - PHGK cells treated with *P. gingivalis* W83 ⟶ ↑ TLR and NLR signalling, apoptosis, inflammatory processes, the NF-κB pathway and the MAPK downstream signalling
Milward et al. [83]	- *P. gingivalis* (ATCC 33277) and *F. nucleatum* (ATCC 10953) strains - Oral epithelial cell OEC line H400 culture - 16 s PCR assays, semi-quantitative reverse transcriptase-polymerase chain reaction (sq-RT-PCR), immunocytochemical staining, microarray	- The H400 cells infected with the heat-killed bacteria ⟶ ↑ TLRs (*TLR-2, -4, -9*) and ↑ NF-κB pathway (*NF-κB1*, *NF-κB2*, *NF-κB1-ε*, *IκB-α*, and *IκB-β*) genes - Infection with *P. gingivalis* and/or *F. nucleatum* ⟶ ↑ TNF-α, IL-1β, IL-8, MCP-1/CCL2, and GM-CSF and cytokeratins 10, 13, and 16 - Infection with *P. gingivalis* and/or *F. nucleatum* ⟶ ↓ cytokeratins 4 and 19 - Infection with *P. gingivalis* and/or *F. nucleatum* ⟶ differential expression of heme oxygenase-1, lysyl oxidase, SOD2, CCL20, and calprotectin
Groeger (2) et al. [85]	- The human squamous cell carcinoma cell lines SCC-25 and BHY - *P. gingivalis* strains W83 and ATCC 33277 - Primary human gingival keratinocytes (PHGK) - Immunostaining, real-time PCR (RT-PCR)	- Expression in SCC-25 and BHY cell lines after infection with *P. gingivalis* W83 ⟶ ↑ B7-H1 and B7-DC receptors - Expression in PHGK after infection with *P. gingivalis* W83 ⟶ ↑ B7-H1 and B7-DC receptors
Groeger (3) et al. [86]	- The human squamous cell carcinoma cell line SCC-25 - Primary human gingival keratinocytes (PHGK) - SCC-25 and PHGK stimulated with total, inner and outer membrane fractions of *P. gingivalis*, cytosolic proteins, as well as LPS and peptidoglycans - Western blot analysis and RT-qPCR	- The total membrane fraction of *P. gingivalis* ⟶ ↑ B7-H1 (PD-L1) expression, followed by the outer and inner membrane - Cytosolic proteins and LPS did not affect B7-H1 (PD-L1) - ↑ B7-H1 (PD-L1) ⟶ immune evasion of oral carcinomas
Wang (2) et al. [88]	- Wild-type (WT) *P. gingivalis* ATCC 33277 and the isogenic Δ*fimA* mutant - Telomerase-immortalized gingival epithelial cells (TIGKs) - Flow cytometry, Western blotting and enzyme-linked immunosorbent assay	- Stimulation with *P. gingivalis* ⟶ ↑ ROS - ROS levels elevated through *P. gingivalis* invades TIGKs in a fimbria (FimA)-dependent manner - The fimbria-deficient mutant of *P. gingivalis* ⟶ no difference in ROS vs. the parental strain
Diomede et al. [89]	- Periodontal ligament biopsies (hPDLSCs) - hPDLSCs divided into untreated control (hPDLSCs) and treated with LPS-G - MTT assay, immunofluorescence analysis, Western blot analysis, next-generation sequencing (NGS)	- hPDLSCs and LPS-G treated hPDLSCs ⟶ ↑ fibroblastic-like shape, ↑ the proliferation rate and ↓ in cell viability - In LPS-G-treated hPDLSCs ⟶ ↑ nuclear translocation of NF-kB, ↑ nuclear histone acetyl transferase p300, and ↓ DNMT1 expression - In control untreated cells ⟶ ↑ cytoplasmic expression of NF-kB and ↑ DNMT1 expression - In LPS-G-treated hPDLSCs ⟶ ↑ amyloid beta precursor protein (APP), amyloid beta precursor protein binding protein 2 (APPBP2), interferon gamma receptor 1 (IFNGR1), matrix metallopeptidase 1 (MMP1), MMP2 and MMP16 vs. control
de Camargo Pereira et al. [91]	- Primary gingival fibroblast and keratinocyte (HaCaT) and gingival fibroblasts cultures - Biopsies of gingival samples from healthy and periodontitis-affected individuals - Medium containing *P. gingivalis* LPS or *P. gingivalis* LPS vehicle - MTT test and quantitative reverse transcription PCR (qRT-PCR)	- *P. gingivalis* LPS ⟶ ↓ DNA methyltransferase 1 (*DNMT1*), DNA methyltransferase 3a (*DNMT3a*), histone demethylases Jumonji domain containing 3 (*JMJD3*) gene expression in HaCaT cells, but no modulation was observed in gingival fibroblasts - No differences in the gene expression analysis in healthy and periodontitis-affected gingival samples
Chang et al. [93]	- OSCC Tca8113 cells infected by *P. gingivalis* at a multiplicity of infection (MOI) of 50 - Non-infected control cells - Western blot analysis and RT-qPCR	- ↑ cell proliferation ⟶ in OSCC Tca8113 vs. control cells - ↑ cells in S phase and ↓ cells in G1 phase ⟶ in OSCC Tca8113 vs. control cells - *P. gingivalis*-infected Tca8113 cells ⟶ of *AP-1*; *c-Jun* and *c-Fos* and *cyclin D1* genes vs. controls cells - ↑ miR-21 and in ↓ PDCD4 in Tca8113 cells infected by *P. gingivalis* - *P. gingivalis* ⟶ OSCC proliferation by regulating cyclin D1 expression via the miR-21/PDCD4/AP-1 negative feedback signalling pathway
Geng et al. [95]	- *P. gingivalis* ATCC 33277 strain - Human immortalized oral epithelial cells (HIOECs)-HIOECs-Pg-15 and HIOECs-Pg-23 (15 and 23 weeks) - Non-infected control - Cell proliferation assay by MTT, cell immunocytochemistry assay, microarray, iTRAQ-based quantitative proteomic assay, quantitative real-time PCR (qRT-PCR), Western blot analysis	- HIOECs-Pg-15 and HIOECs-Pg-23 ⟶ anomalous shapes with absent contact inhibition; the rich and thick tonofilaments in infected cells - Infected HIOECs ⟶ greater proliferation ability vs. control - The number of S phase cells, cell migration and invasion abilities ⟶ HIOECs-Pg-15 and HIOECs-Pg-23 vs. HIOECs - In HIOECs-Pg-15 ⟶ ↑ *CXCL10*, *CSF1*, *IL-6*, *NNMT*, *CYGB*, *CXCL11*, *FLI1*, *WFDC2*, *CCAT1*, *CD274*, and *PDCD1LG2* genes vs. control - In HIOECs-Pg-23 ⟶ ↑ *CSF1*, *NNMT*, *CYGB*, *FLI1*, *GAS6*, *CCAT1*, *CD274*, and *PDCD1LG2* genes vs. control
Zhou et al. [96]	- *P. gingivalis* W83, ATCC 33277 (33277) strains, and the Δ*fimA*, Δ*rgpAB*, Δ*kgp*, and Δ*rgpAB* Δ*kgp* isogenic 33277 mutants - Telomerase immortalized gingival epithelial keratinocytes (TIGKs) derived from a primary gingival epithelial cell line - Western blot analysis, transfection and TCF/LEF reporter assay, immunofluorescence, quantitative reverse transcriptase PCR (qRT-PCR)	- *P. gingivalis* ⟶ cleavage of β-catenin and GSK3β - *P. gingivalis* ⟶ stability of β-catenin, but at multiplicities of infection ⟶ partial degradation of β-catenin - *P. gingivalis*-dependent degradation of GSK3β ⟶ disruption of the β-catenin destruction complex - Proteolysis of β-catenin and GSK3β is related to the gingipain proteases (RgpA and RgpB) and not a consequence of increased proteasomal activity in the host cells - Mutants of *P. gingivalis* deficient in gingipain production ⟶ ↓ cleavage of β-catenin and GSK3β
Kuboniwa et al. [97]	- *P. gingivalis* 33277 and YPF1 (FimA^−^) - Primary cultures of gingival epithelial cells (GECs) - Flow-cytometry, cell cycle analysis with CellQuest and ModFit LT V 3.1 software, microarrays	- *P. gingivalis* modulates the activation of cell cycle control proteins, i.e., cyclins, p53 and PI3K - *P. gingivalis* ⟶ ↑ Cyclin A, Cdk4, Cdk6 and PI3K and ↓ INK4 and Cyclin D and p53 - *P. gingivalis* ⟶ ↑ progression through the S phase - ↑ GECs proliferation related to the presence of long fimbriae of *P. gingivalis*
Cho et al. [99]	- *P. gingivalis* 381 strain - The oral cancer cells, SCC-25 and Ca9-22 - Non-infected control - MTT assay, cell cycle, proliferative activity, and autophagic response analysis - ROS generation detected by DCFDA assay	- *P. gingivalis* ⟶ lack of SCC-25 and Ca9-22 cytotoxicity-induced. - SCC-25 and Ca9-22 oral cancer cells exhibited reduced proliferation after *P. gingivalis* infection by inducing G1 cell cycle arrest - *P. gingivalis* ⟶ no effect on apoptosis - *P. gingivalis* ⟶ ↓ cyclin D1 and Cdk4 and ↑ level of p21, and Cdk inhibitor, vs. non-infected controls - Autophagic response was activated by the formation of ROS
Choi et al. [100]	- *P. gingivalis* ATCC 33277 strain - Primary gingival epithelial cells (GECs) - HIGK (human immortalized gingival keratinocytes) cell line - Measurement of ROS production (MitoSOX Red), cytofluorimetry, generation of P2X_7_ and pannexin-1 knockdown epithelial cells (Western-blot analysis), luminescence-based GSH/GSSG-Glo Assay, quantitative PCR, ATP hydrolysis assay, LDH viability assay, extracellular ATP release assay	- Extracellular ATP (eATP) ↑ the cellular ROS levels in GECs vs. untreated control cells - P2X_7_ receptor signalling coupled with NADPH-oxidase activation ⟶ ↑ ROS - *P. gingivalis* GECs infection ⟶ ↑ the antioxidant glutathione response, modulated eATP-induced cytosolic and mitochondrial ROS generated the through P2X_7_/NADPH-oxidase interactome - *P. gingivalis* effector, nucleoside-diphosphate- kinase (Ndk) ⟶ ↓ oxidative-stress in GECs - *P. gingivalis* infection ⟶ ↑ anti-oxidative mitochondrial UCP_2_ levels - *Ndk*-deficient *P. gingivalis* mutant lacked the ability to inhibit ROS production
Roberts et al. [101]	- *P. gingivalis* ATCC strain 33277 - Primary gingival epithelial cells (GECs) - Quantitative real-time PCR (qPCR) using specific TaqMan primers, the NADP/NADPH assay, epifluorescence (DM IRE2 HC inverted scope), immunoprecipitation, immunoperoxidase staining, ELISA, MPO activity assay (fluorometric), SybrGreen quantitative real-time PCR	- *P. gingivalis* ⟶ ↓ eATP/P2X7 signalling, i.e., the generation of ROS and NADPH oxidases (NOX) from primary GECs - *P. gingivalis* infected human primary GECs ⟶ eATP stimulation increased the mRNA expression of NOX2 - *P. gingivalis* ⟶ reorganizing the localization and activation of cytosolic molecules (p47phox, p67phox, and Rac1) - *P. gingivalis* ⟶ ↓ the MPO product-bactericidal HOCl upon eATP stimulation - *P. gingivalis* ⟶ ↓ glutathione and ↑ glutamate cysteine ligase (GCL) subunits GCLc and GCLm, glutathione synthetase, and glutathione reductase
Mao et al. [103]	- *P. gingivalis* strains ATCC 33277, ATCC 49417, W83 and A7A1-28, YPF1 (fimA^−^), SMF1 (mfa^−^) - Primary gingival epithelial cells (GECs) - ELISA-based detection of histone associated DNA fragments, caspase-3 (DEVDase) activity assay, Western immunoblotting, Real-time quantitative PCR (rRT-PCR)	- *P. gingivalis* infection of GECs ⟶ the phosphorylation of JAK1 and STAT3 - *P. gingivalis-infected* GECs ⟶ ↑ Survivin and STAT3 - *P. gingivalis* ⟶ blockage of apoptotic pathways in GECs through the JAK1/STAT3 pathway - *P. gingivalis* ⟶ ↓ camptothecin-induced activation of caspase-3 in a dose-dependent manner, but regardless of both the long (FimA) and short (Mfa) fimbriae
Yilmaz (1) et al. [104]	- *P. gingivalis* ATCC 33277 strain - Primary gingival epithelial cells (GECs) - Infection of cells with *P. gingivalis* and treatment with zVAD-fmk, staurosporine, and PI3K inhibitor - Flow cytometry, immunoblot analysis, fluorescence microscopy, TUNEL assay	- *P. gingivalis* ⟶ rapid and reversible surface phosphatidylserine exposure through a mechanism requiring caspase activation - *P. gingivalis* ⟶ ↓ depolarization of the mitochondrial transmembrane potential and cytochrome c release in GECs - Suppression of the PI3K/Akt pathway following staurosporine and PI3K inhibitor (LY294002) treatment ⟶ mitochondrial membrane depolarization, cytochrome c release, DNA fragmentation, and increased apoptosis of infected GECs
Nakayama et al. [105]	- The human gingival epithelial Ca9-22 cell line - *P. gingivalis* wild-type strain ATCC3327 (WT) and the gingipains-deficient mutant strain KDP136 (*rgpA*, *rgpB*, and *kgp*) - Western blotting, immunofluorescence analysis, transwell assay, pulldown assay for biotin-labelled membrane proteins	- Live *P. gingivalis* infection live, but not heat-killed *P. gingivalis* ⟶ ↑ dephosphorylation of Akt at infection time-dependent manner - *P. gingivalis* ⟶ Akt inactivation and ↑ dephosphorylation of GSK3α/β, mTOR, and Bad - invading *P. gingivalis* Ca9-22 cells or endocytosed virulence factors from *P. gingivalis* are not associated with Akt dephosphorylation by *P. gingivalis*
Nakhjiri et al. [106]	- *P. gingivalis* 33277 cells - Primary gingival epithelial cells (GECs) - Non-infected controls - DNA fragmentation ELISA assay, immunoblotting	- *P. gingivalis* ⟶ ↑ in GEC DNA fragmentation, but prolonged incubation GECs did not undergo apoptosis - *P. gingivalis* ⟶ ↑ blockage of apoptosis in GECs following stimulation with camptothecin - *P. gingivalis* ⟶ ↓ apoptosis in GECs by up-regulation of the anti-apoptotic molecule Bcl-2
Moffatt et al. [107]	- *P. gingivalis* 33277 cells - Primary gingival epithelial cells (GECs) - Non-infected controls - miRNA array, quantitative RT-PCR (qRT-PCR) of miR-203, quantitative RT-PCR for mRNA, dual luciferase reporter assay, Western immunoblotting	- *P. gingivalis* ⟶ ↑ miR-203 in GECs (upregulated 4-fold) vs. uninfected controls - *P. gingivalis*-infected GECs ⟶ ↓ SOCS3 and SOCS6 mRNA levels (5-fold and 2-fold, respectively) - ↑ miR-203 levels in GECs ⟶ ↓ SOCS3 (miR-203 binds the 3′ UTR region of SOCS3) - SOCS3 mRNA levels by *P. gingivalis* is mediated by miR-203
Yilmaz (2) et al. [110]	- *P. gingivalis* 33277 cells - Primary gingival epithelial cells (GECs) - Non-infected controls - Reverse transcription PCR (RT-PCR) analysis, immunofluorescence microscopy, cytofluorimetry, TUNEL assay, fluorescence microscopy	- *P. gingivalis* ⟶ ↓ GECs apoptosis induced by ATP ligation of P2X_7_ receptors - A *P. gingivalis* homologue of nucleoside diphosphate kinase (NDK) ↓ GECs apoptosis - NDK ⟶ ↑ survival of host cells by hydrolysing extracellular ATP (eATP) and ↓ apoptosis-mediated through P2X_7_ - *P. gingivalis* infection suppressed considerably the pro-apoptotic effect of ATP
Ohshima et al. [112]	- *P. gingivalis* ATCC 33277, W83, low passage strain MP4-504, ΔrgpAB, Δkgp, and ΔrgpAB/kgp - Human telomerase immortalized gingival keratinocytes (TIGKs) and OKF6/TERT2 keratinocytes - SCC9 squamous carcinoma cells derived from the tongue - Non-infected controls - Quantitative reverse transcription-PCR (qRT-PCR), immunoblotting, chromatin immunoprecipitation, immunofluorescence, matrigel invasion assay	- *P. gingivalis* ⟶ ↑ ZEB2 mRNA levels in TIGK cells in a time- and dose-dependent manner - In contrast, *P. gingivalis* had a less pronounced effect on TWIST1/2 mRNA - While *P. gingivalis* remained capable of increasing ZEB2 transcripts in the presence *F. nucleatum*, coinfection with *S. gordonii* antagonized induction of ZEB2 by *P. gingivalis* - Knockdown of ZEB2 with siRNA ⟶ ↓ TIGK migration into matrigel and IL-6 mRNA production in response to *P. gingivalis* - Wnt/β-Catenin and FOXO1 signalling controls ZEB2 expression in response to *P. gingivalis*.
↑ higher expression/activity; ↓ lower expression/activity		

**Table 2:** The effect of *P. gingivalis* infection on oral epithelial/cancer cells in the selected animal model and clinical studies.

Author	*P. gingivalis* in Animal Model and Clinical Studies of OSCC	
	Study Design and Methodology	Key Findings
Animal model studies		
Gallimidi et al. [76]	- Experimental system that combines the mouse model of chronic periodontitis with the carcinogen 4-nitroquinoline-1-oxide (4NQO)-induced oral carcinoma model - Mice were administered 4NQO in the drinking water for 8 weeks - The 4NQO-treated mice were repeatedly infected with a mixture of two periodontal pathogens, *P. gingivalis* and *F. nucleatum* every other day, initiated 2 weeks prior to 4NQO administration, and continued (2 times/week) until week 18 - Bacteria were recovered from the tongue surface by swabbing and culturing - H&E staining, morphometric and immunohistochemical analysis, quantitative RT-PCR analysis (qRT-PCR)	- Tumours from infected mice were 2.5 times larger and were significantly more invasive vs. non-infected mice - Infected mice ⟶ ↑ cyclin D1 vs. non-infected mice, both in cancerous and non-cancerous tongue epithelium - Three consecutive administrations of *P. gingivalis*/*F. nucleatum* ⟶ ↑ STAT3 in the tongue epithelium (nuclear-localized pSTAT3) in infected vs. non-infected mice - In the tongue mucosa of infected mice ⟶ ↑ *IL-6* mRNA level
Sztukowska et al. [77]	- BALB/c mice orally infected with 10^7^ cfu *P. gingivalis* 33277 five times at 2-day intervals - The levels of *P. gingivalis* colonization were determined by qPCR with the *P. gingivalis* 16SrRNA gene - On days 1, 3, and 8 after the last infection, mice were euthanized and the upper and lower jaw with gingival tissue were recovered. - The ratio of *ZEB1* mRNA was determined by qRT-PCR	- Colonization with *P. gingivalis* ⟶ ↑ *ZEB1* mRNA at days 1, 3, and 8 after infection compared to sham infected animals
Yao et al. [113]	- Eight-week-old Balb/c male mice - All mice were randomized into two groups: one group was colonized by *P. gingivalis* and *F. nucleatum* (P+) and another served as control - For P+ group, 200 μL of a mix of bacteria was applied at the surface of the mandibular molar teeth, four times a week, for 1 month - 5 × 10^6^ SCC-7 cells were injected into the submucosa of the right cheek after 3 weeks of bacterial colonization - After the tumour inoculation, the diameter of the mass was measured in three directions, and primary tumour growth or formation was evaluated for 3 weeks - Western blot, histological examination, immunostaining, and real-time PCR	- ↑ The tumour mass and growth rate in the P+ group vs. control group - Tumour tissues ⟶ flaky necrosis, with lots of damaged vascular profile and cell debris - In tumour tissues of the P+ group ⟶ ↑ Ki67 and cyclin D1 - Periodontitis-associated bacteria ⟶ ↑ IL-6, TNF-α, IL-18, ASC (up to 6 times), and caspase-1 (up to 4 times) - Periodontitis-associated bacteria ⟶ ↓ NF-κB, NOD-, LRR- and NLRP3, and IL-1β (less than 0.5 times) - In the P+ group ⟶ ↑ CD4+ T cells, CD8+ T cells, and CD206+ macrophages - In tumour tissues ⟶ ↑ γ-H2AX, p-ATR, RPA32, CHK1, and RAD51, and ↓ the phosphorylation level of CHK1 (p-chk1)
Clinical studies		
Al-hebshi et al. [115]	- Characteristics of the species composition as well as functional potential of the bacteriome associated with OSCC - DNA extraction from 20 fresh OSCC biopsies (cases) and 20 deep-epithelium swabs (matched control subjects) - DDK DNA isolation kit - BLASTN-algorithm, QIIME, PICRUSt, and LEfSe analyses	- The most overrepresented species in the tumours ⟶ *F. nucleatum*, but also *P. gingivalis* - The most overrepresented species in the controls ⟶ *S. mitis*, *R. mucilaginosa* and *H. parainfluenzae* - In the tumours ⟶ ↑ genes involved in bacterial mobility, flagellar assembly, bacterial chemotaxis and LPS synthesis - In the tumours ⟶ ↑ genes encoding antibiotic transport system permease and ATP binding proteins, 7,8-dihydro-8-oxoguanine-triphosphatase and ABC-2 type transport system permease and ATP binding proteins - In the controls ⟶ ↑ genes responsible for DNA repair and combination, purine metabolism, phenylalanine, tyrosin and tryptophan biosynthesis, ribosome biogenesis and glycolysis/gluconeogenesis - In the controls ⟶ ↑ genes encoding methyl accepting chemotaxis protein, restriction enzyme subunits and peptide nickel transport system permease and ATP binding proteins
Zhang et al. [116]	- Characteristics of the species of in the human microbiome in OSCC - Comparison of the microbiota compositions between tumour sites and opposite normal tissues as a control - Oral buccal mucosa of 50 patients with OSCC - DNA extraction, polymerase chain reaction (PCR) amplification, and 16S rRNA gene sequencing	- In tumour sites ⟶ ↑ richness and diversity of bacteria vs. the control tissues. - In cancer tissues ⟶ 6 families and 13 genera, including *P. gingivalis* and *F. nucleatum* - In the tumours ⟶ ↑ genes involved in bacterial chemotaxis, flagellar assembly and lipopolysaccharide (LPS) biosynthesis
Chang et al. [117]	- Subgingival plaque, cancer and paracancerous tissues from six patients with OSCC - 16S rRNA amplicon sequencing, qPCR and fluorescence in situ hybridization	- In 61 cancer tissues, paracancerous tissues and subgingival plaque samples ⟶ ↑ *P. gingivalis*, *F. nucleatum* vs. detection in 30 normal tissues - *P. gingivalis* infection ⟶ in 60.7% of OSCC tissues, 32.8% of paracancerous tissues and 13.3% of normal tissues - *P. gingivalis* infection ⟶ ↑ late clinical staging, low differentiation and lymph node metastasis in patients with OSCC
Yost et al. [118]	- Pilot study of community-wide metatranscriptome analysis to profile mRNA expression in the entire oral microbiome in OSCC - A cross-sectional comparison of gene expression in subjects with and without OSCC - Oral swab samples collected from four distinct sites: from the OSCC tumour site, a healthy control site from a healthy patient matching the tumour site, a healthy buccal site from a tumour-free healthy individual and an OSCC tumour-adjacent site (all from buccal sites) from a cancer patient - Identification of the expressed genes using metatranscriptome analyses (NOISeq and GFOLD)	- In cancer tissue ⟶ ↑ number of transcripts at tumour sites and tumour-adjacent sites of cancer patients vs. the healthy controls - In cancer tissue ⟶ ↑ iron ion transport, tryptophanase activity, peptidase activities protease activity (zinc metalloproteases, ATP-dependent Clp protease, serine protease and immunoglobulin protease) and superoxide dismutase were over-represented in tumour and tumour-adjacent samples vs. the healthy controls - In cancer tissue ⟶ ↑ capsule biosynthesis, flagellum synthesis and assembly (FlgG, FliL and flagellar MS-ring protein), chemotaxis (cheR and cheV), iron transport (ferrous iron transporters, iron transport systems and yersiniabactin), haemolysins and adhesins - In non-tumour sites of cancer ⟶ ↑ protection against reactive nitrogen intermediates, chemotaxis, flagellar and capsule biosynthesis
Katz et al. [52]	- Investigation of the presence of *P. gingivalis* in specimens from squamous cell carcinoma patients - immunohistochemical staining (IHC)	- Staining for *P. gingivalis* ⟶ ↑ levels in gingival carcinoma vs. in normal gingival tissues (more than 33%)
Li et al. [119]	- The microbial composition and functions in periodontitis and gingival squamous cell carcinoma (GSCC) - GSCC patients (*n* = 10), matched periodontitis patients (*n* = 15), and healthy individuals (*n* = 15) - Saliva, subgingival plaque, tongue dorsum, buccal mucosa, cancerous tissue, and paracancerous tissue samples - 16S rDNA amplicon sequencing and the taxonomic analysis	- Infection with periodontal pathogens ⟶ 46% in GSCC, 38.36% in the subgingival plaque and 44.13% from saliva - In cancerous tissues ⟶ ↑ *Fusobacterium* spp., *Peptostreptococcus* spp., and *Prevotella* spp. - In saliva and subgingival plaque ⟶ ↑ *Atopobium* in GSCC vs. periodontitis and controls - In subgingival plaque of GSCC ⟶ ↑ lipopolysaccharide (LPS) biosynthesis vs. buccal mucosa, and paracancerous tissue samples
Hou et al. [120]	- Oropharyngeal mucosa (with oral mucosistis-complications of radiation therapy) of patients were examined regularly, and sampled longitudinally in eight stages of their radiation treatment programme: before radiation, and then after 10, 20, 30, 40, 50, 60, and 70 Gy - 16S rRNA gene sequencing and bioinformatics analysis	- The mucosal bacterial diversity did not change during the entire course of these patient treatments - Necrotizing ulcerative gingivostomatitis ⟶ ↑ *Prevotella* spp., *Fusobacterium* spp., and *Porphyromonas* spp. - Dynamic synchronous variations in abundances of bacteria throughout the course of radiation therapy - Peaks frequently coincided with the onset of severe mucositis
↑ higher expression/activity; ↓ lower expression/activity		

## Data Availability

Not applicable.

## Abbreviations

The following abbreviations are used in this manuscript:

BCL-2	B-cell lymphoma 2
CCND1	Gene encodes the cyclin D1 protein
CDKs	Cyclin-dependent kinases
CDKN2A	Cyclin-dependent kinase inhibitor 2A
DNMT	DNA methyltransferase 1
eATP	Extracellular ATP
EGFR	Epidermal growth factor receptor
EMT	Epithelial-to-mesenchymal transition
FGFR	Fibroblast growth factor receptors
GSK3β	Glycogen synthase kinase 3 beta
hTERT	Human telomerase catalytic subunit
hPDLSCs	Human periodontal ligament stem cells
HRAS	GTPase, also known as transforming protein p21
HNSCC	Head and neck squamous cell carcinoma
HPV	Human papillomavirus
JAK1/2	Janus kinase 1/2
Ltp1	Low-molecular-weight tyrosine phosphatase
LOX	Lysyl oxidase
ILs	Interleukins
LPS	Lipopolysaccharide
FimA/Mfa1	Fimbriae
RgpA/RgPb	Gingipains (cysteine proteinases)
OMVs	Outer membrane versicles (OMVs)
HA	Hemagglutinins
HM0 × 1	Heme oxygenase-1
MiRNA	Micro ribonucleic acid
MAPK	Mitogen-activated protein kinase
MMPs	Matrix metalloproteinases
MPO	Myeloperoxidase
mTOR	Mammalian target of rapamycin
NDK	Nucleoside-diphosphate-kinase
NLRP3	Pyrin domain-containing protein 3
NF-κB	Nuclear factor kappa beta
NOD1	Nucleotide-binding oligomerization domain protein 1
NOTCH1	Neurogenic locus notch homologue protein 1
OMVs	Outer membrane versicles
OPSCC	Oropharynx squamous cell carcinomas
OSCC	Oral squamous cell carcinoma
OTSCC	Tongue squamous cell carcinoma
PIK3CA	Phosphatidylinositol-4,5-bisphosphate 3-kinase
PPAD	Citrullinating enzyme—peptidylarginine deiminase
PDCD4	Programmed cell death 4
RB1	Retinoblastoma protein, a tumour suppressor protein
STAT3	Signal transducer and activator of transcription 3
SOD2	Superoxide dismutase 2
TLRs	Toll-like receptors
TIGKs	Gingival epithelial keratinocytes
TP53	Tumour protein p53
TNF	Tumour necrosis factor
